# MicroRNA Expression and Regulation in Human, Chimpanzee, and Macaque Brains

**DOI:** 10.1371/journal.pgen.1002327

**Published:** 2011-10-13

**Authors:** Hai Yang Hu, Song Guo, Jiang Xi, Zheng Yan, Ning Fu, Xiaoyu Zhang, Corinna Menzel, Hongyu Liang, Hongyi Yang, Min Zhao, Rong Zeng, Wei Chen, Svante Pääbo, Philipp Khaitovich

**Affiliations:** 1 Key Laboratory of Computational Biology, CAS–MPG Partner Institute for Computational Biology, Chinese Academy of Sciences, Shanghai, China; 2 Key Laboratory of Systems Biology, Institute of Biochemistry and Cell Biology, Shanghai Institutes for Biological Sciences, Chinese Academy of Sciences, Shanghai, China; 3 College of Life Science, Northeast Forestry University, Harbin, China; 4 Max Planck Institute for Molecular Genetics, Berlin, Germany; 5 Max Delbrück Center for Molecular Medicine, Berlin Institute for Medical Systems Biology, Berlin-Buch, Germany; 6 Max Planck Institute for Evolutionary Anthropology, Leipzig, Germany; University of Illinois at Urbana-Champaign, United States of America

## Abstract

Among other factors, changes in gene expression on the human evolutionary lineage have been suggested to play an important role in the establishment of human-specific phenotypes. However, the molecular mechanisms underlying these expression changes are largely unknown. Here, we have explored the role of microRNA (miRNA) in the regulation of gene expression divergence among adult humans, chimpanzees, and rhesus macaques, in two brain regions: prefrontal cortex and cerebellum. Using a combination of high-throughput sequencing, miRNA microarrays, and Q-PCR, we have shown that up to 11% of the 325 expressed miRNA diverged significantly between humans and chimpanzees and up to 31% between humans and macaques. Measuring mRNA and protein expression in human and chimpanzee brains, we found a significant inverse relationship between the miRNA and the target genes expression divergence, explaining 2%–4% of mRNA and 4%–6% of protein expression differences. Notably, miRNA showing human-specific expression localize in neurons and target genes that are involved in neural functions. Enrichment in neural functions, as well as miRNA–driven regulation on the human evolutionary lineage, was further confirmed by experimental validation of predicted miRNA targets in two neuroblastoma cell lines. Finally, we identified a signature of positive selection in the upstream region of one of the five miRNA with human-specific expression, miR-34c-5p. This suggests that miR-34c-5p expression change took place after the split of the human and the Neanderthal lineages and had adaptive significance. Taken together these results indicate that changes in miRNA expression might have contributed to evolution of human cognitive functions.

## Author Summary

Humans are remarkably similar to apes and monkeys on the genome sequence level but remain remarkably distinct with respect to cognitive abilities. How could human cognition evolve within such a short evolutionary time? Among many hypotheses, evolution in expression of a few key regulators affecting hundreds of their target genes was proposed as one possible solution. Here, we tested this notion by studying expression divergence of a specific type of regulatory RNA, microRNA (miRNA), and its effect on gene expression profiles in brains of humans, chimpanzees, and rhesus macaques. Our results indicate that changes in miRNA expression have played a considerable role in the establishment of gene expression divergence between human brains and brains of non-human primates at both mRNA and protein expression levels. Furthermore, we find indications that some of the human-specific gene expression profiles caused by miRNA expression divergence might be associated with evolution of human-specific functions.

## Introduction

Phenotypic differences between species, including human-specific features such as language and tool-making, are thought to have arisen, to a large extent, through changes in gene expression [1]. Indeed, humans and the closest living primate relatives, chimpanzees, display substantial gene expression divergence in all tissues including the brain [2], [3]. Mechanistically, this divergence might have been caused by mutations in regulatory elements proximal to genes (*cis-* effects), or changes in expression or sequence of distal regulators (*trans-* effects). Previous studies focusing on transcription factors (TFs) have indicated an excess of human-specific expression divergence for several TFs in the liver [4] and the brain [5]. These findings suggest that changes in TF expression might explain some of human-chimpanzee gene expression divergence.

In this study, we investigated the contribution of another type of gene expression regulator, miRNA, to human-specific gene expression divergence. miRNA are short (20–23-nucleotide), endogenous, single-stranded RNA involved in post-transcriptional gene expression silencing. Mature miRNA function as part of the RNA-induced silencing complex (RISC), mediating post-transcriptional gene expression inhibition [6]–[8]. In animals, the predominant mechanism of miRNA-mediated gene silencing employs complementary base-pairing between the miRNA seed region and the mRNA 3′ UTR region [9], [10]. This interaction guides RISC to target transcripts, which are consequently degraded, destabilized or translationally inhibited, causing an inverse expression relationship between miRNA and its cognate targets [8]–[12]. miRNA-mediated gene expression silencing has previously been shown to be important for a variety of physiological and pathological processes, ranging from developmental patterning to cancer progression, as well as important neural functions and dysfunctions [7], [13]–[15]. The roles of miRNA in determining gene expression divergence between species and, in particular, their contribution to expression differences specific to the human brain remains, however, largely unknown.

## Results/Discussion

### Estimating miRNA Expression Divergence by High-Throughput Sequencing

To assess miRNA expression divergence between human brains and brains of closely related primate species, we measured miRNA levels in two distinct brain regions, the prefrontal cortex (dorsal-lateral prefrontal region) and the cerebellum (lateral cerebellar cortex), of humans (age: 14–58 years), chimpanzees (age: 12–40 years) and rhesus macaques (age: 6–15 years) using high-throughput sequencing (Illumina).

In the prefrontal cortex, a brain region known to play a part in the control of high-level cognitive functions, such as abstract thinking and planning [16]–[18], we measured miRNA expression samples containing RNA pooled from multiple individuals, for each species (Table S1). To assess technical variation of the sequencing measurements, we prepared and sequenced small RNA libraries twice. In the cerebellum, we sequenced two human samples, one chimpanzee sample and one rhesus macaque sample, all composed from RNA pooled from multiple individuals (Table S1).

We obtained an average of 7.6 million sequencing reads per sample, approximately 49% of which could be perfectly mapped to the corresponding reference genome (Table S2). Based on these data, we detected expression of 413 miRNA covered by least 10 sequence reads in the human prefrontal cortex or cerebellum. To obtain the corresponding miRNA expression estimates for chimpanzees and rhesus macaques, we mapped all annotated human miRNA precursors to the chimpanzee and rhesus macaque genomes, using a combination of reciprocal BLAT, BLAST and liftOver [19]–[21] and extracted mature miRNA sequences using ClustalW2 precursor sequence alignment [22] (Materials and Methods). For 413 miRNA expressed in the human brain, we could unambiguously identify 385 and 390 corresponding genomic locations in the chimpanzee and rhesus macaque genomes, respectively. The vast majority of these miRNA were also detected in chimpanzee (375) and rhesus macaque (366) brains (Table S3). Due to lower quality of the chimpanzee and the rhesus macaque genomes as well as low expression levels of human miRNA with no chimpanzee or macaque orthologs, we omitted these miRNA from further analyses.

**Figure 1:**
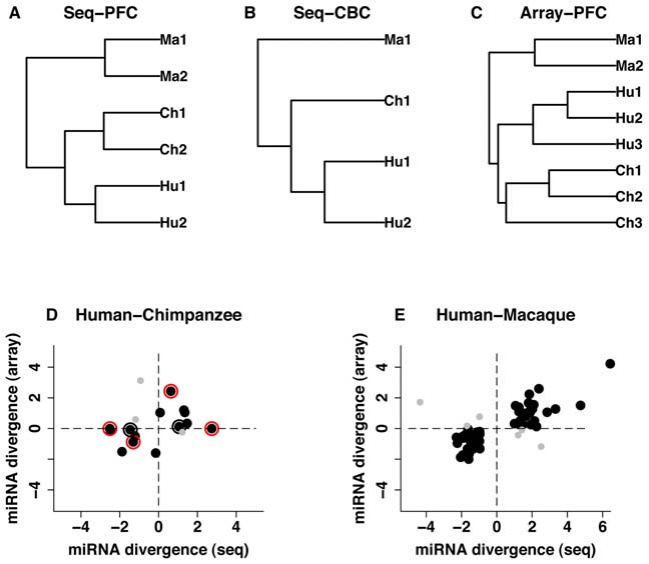
miRNA expression divergence among species and between two brain regions. A-C, UPGMA dendrograms based on miRNA expression measurements detected in humans (Hu), chimpanzees (Ch) and rhesus macaques (Ma) in at least one sample: (A) prefrontal cortex, high-throughput sequencing (*N* = 572); (B) cerebellum, high-throughput sequencing (*N* = 539); and (C) prefrontal cortex, microarrays (*N* = 325). D-E, miRNA expression divergence (log2-transformed fold-change) measured using microarrays (Array) and high-throughput sequencing (Seq). (D) miRNA with significant expression divergence between human and chimpanzee prefrontal cortex identified using at least one of the two methodologies (*N* = 17, fifteen miRNA detected by both microarrays and sequencing, two [miR-184 and miR-299-3p] – detected by sequencing and verified by Q-PCR). (E) miRNA with significant expression divergence between human and rhesus macaque prefrontal cortex (*N* = 61). The black dots indicate miRNA showing consistent expression change directions in the two methodologies; grey dots – miRNA showing inconsistent directions of expression changes; red outer circles – miRNA expression differences confirmed using Q-PCR; black outer circles – unconfirmed miRNA expression differences.

In all three species, high-throughput sequencing generated highly reproducible miRNA expression measurements, with good positive correlation between technical replicates (Pearson correlation, *r*>0.99, *p*<10^−15^) (Figure S1). Furthermore, in both brain regions, miRNA expression divergence among species was evidently greater than variation within species (Figure 1A-1B). The extent of miRNA expression divergence followed the phylogenetic relationship among species in both prefrontal cortex and cerebellum, *i.e.* human and chimpanzee samples clustered as sister species, with macaque samples forming an outgroup.

In the human or chimpanzee prefrontal cortex, 325 miRNA were represented by at least 10 sequence reads in at least one technical replicate of one species. All 325 miRNA had orthologs in the chimpanzee genome (Table S3). Of these, 37 were differently expressed between species in both technical replicates (Fisher's exact test, *p*<0.01 & fold-change>2). Using an alternative procedure, based on the assumption that sequence read follow a negative binomial distribution, implemented in the edgeR package [23], 35 miRNA were differently expressed between humans and chimpanzees (*p*<0.001 & FDR<0.01) (Table S4). Thirty one overlapped between the two methods (binomial test, *p*<0.0001). Using the same criteria, 106 out of 338 miRNA detected in human and rhesus macaque prefrontal cortex were differently expressed between the two species, according to Fisher's test. Eighty-eight out of these 106 miRNA were also classified by edgeR as differently expressed (Table S4).

The vast majority of miRNA expression differences that were found between species in the prefrontal cortex could be reproduced in the cerebellum. Specifically, out of 37 miRNA differently expressed between humans and chimpanzees in the prefrontal cortex, according to Fisher's exact test, 31 (84%) showed consistent expression differences between species in both brain regions (Figure S2A-S2B). Similarly, out of 106 miRNA differently expressed between humans and macaques in the prefrontal cortex, 82 (77%) showed consistent expression differences between the two species in both brain regions (Figure S2C-S2D). In both cases, the agreement between the two brain regions was far greater than expected by chance (binomial test, p<0.0001).

Although the prefrontal cortex and the cerebellum are histologically different, previous studies have shown that mRNA expression differences between humans and chimpanzees are largely shared between these two brain regions [24]. Our results indicate that miRNA divergence is similarly shared between the prefrontal cortex and the cerebellum. Furthermore, good agreement of miRNA divergence estimates between the two brain regions supports robustness of our measurements.

### Validation of miRNA Expression Divergence Estimates by Microarrays and Q-PCR

To further test the robustness of the miRNA divergence estimates obtained using high-throughput sequencing and to overcome potential problems caused by pooling samples from multiple individuals, we measured miRNA expression in the prefrontal cortex of three human, three chimpanzee and two rhesus macaque individuals using miRNA microarrays (Agilent). To exclude possible hybridization artefacts, array probes corresponding to 150 miRNA with sequence differences between humans and chimpanzees and to 313 miRNA with sequence differences between humans and rhesus macaques were masked prior to further analyses. Overall, microarray were less sensitive than sequencing, with a total of 287 miRNA detected as expressed above the default threshold in the human or chimpanzee prefrontal cortex (Table S5).

Concordant with results obtained using high-throughput sequencing, intra-species variation of the microarray miRNA expression measurements was lower than between-species divergence. Further, as in case of the sequencing data, miRNA expression divergence measured by arrays followed the phylogenetic relationship among species (Figure 1C).

Further supporting the authenticity of our miRNA divergence estimates, the differences found between humans and chimpanzees or macaques, using high-throughput sequencing, were largely reproduced in microarray experiments. Due to the lower sensitivity of microarray experiments, out of 37 miRNA that were classified as differently expressed between human and chimpanzee prefrontal cortex using sequencing, 12 were detected reliably on the microarrays and 9 showed consistent direction of expression divergence (Figure 1D). Analyzing miRNA expression divergence based on microarray data alone and applying statistical criteria similar to the ones used in sequencing data analysis (Student's t-test, *p*<0.01, fold-change>2), four miRNA differed significantly between humans and chimpanzees in the prefrontal cortex. Three of these showed consistent direction of expression change and one passed the significance cutoff level in the sequencing data. Thus, out of 15 miRNA classified as being differently expressed between humans and chimpanzees by at least one methodology, 12 showed consistent direction of expression change (binomial test, *p*<0.05) (Figure 1D). Similarly, out of 106 miRNA differently expressed between human and macaque prefrontal cortex, according to sequencing, 61 were detected by microarrays and 55 showed consistent direction of expression change (binomial test, *p*<0.01) (Figure 1E).

Despite overall agreement between the two methodologies, some of the human-chimpanzee miRNA expression differences identified using sequencing were not confirmed by the microarrays. To further test the validity of our results, we measured expression of 6 miRNA in three human and three chimpanzee individuals using a third methodology: quantitative RT-PCR. We chose three types of miRNA differences: (1) consistent by both methodologies: miR-383 and miR-34c-5p; (2) significant according to sequencing, but unconfirmed in the microarray experiment: miR-143 and miR-499; (3) significant according to sequencing, but not detected or masked on the microarrays: miR-184 and miR-299-3p. Quantitative RT-PCR results confirmed expression differences for all miRNA in the first and the third categories, but not for the miRNA in the second category (Figure S3). Thus, miRNA expression differences that were consistent across methodologies, or large differences that were identified by the sequencing, but masked or undetected on the arrays, are both likely to reflect actual miRNA expression divergence between human and chimpanzee brains.

**Figure 2:**
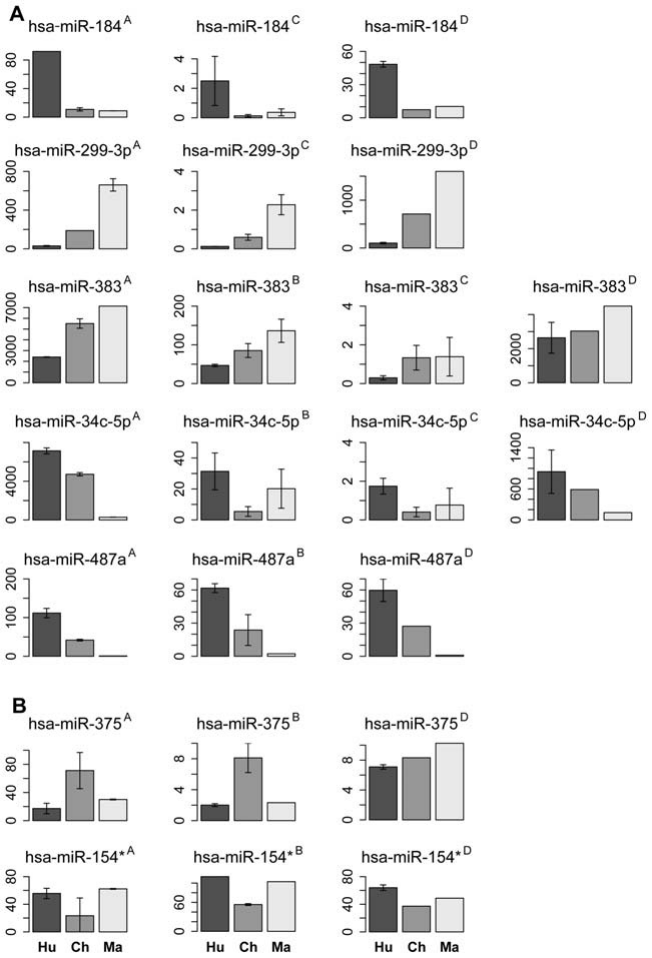
miRNA with species-specific expression in prefrontal cortex. A, miRNA with human-specific expression profiles in prefrontal cortex confirmed by at least two methodologies. B, miRNA with chimpanzee-specific expression profiles in prefrontal cortex confirmed by at least two methodologies. The panel titles show miRNA identity and the measurement methodology: ^A^ - sequencing, ^B^ – microarrays, ^C^ - Q-PCR, or ^D^ - miRNA levels measured using sequencing in cerebellum. The bar colors and labels indicate species: dark grey/Hu – human; grey/Ch – chimpanzee; light grey/Ma – macaque. Note that all miRNA identified as showing species-specific profiles in prefrontal cortex, except miR-375, show analogous species-specific profiles in cerebellum. The expression levels are shown as mean of quantile normalized miRNA reads count for high-throughput sequencing, mean quantile normalized miRNA florescent signal intensities for microarrays or mean Q-PCR cycle numbers normalized to the cycle numbers of invariant internal standard. The error bars show one standard deviation of the measurements.

In the prefrontal cortex, we identified 25 miRNA with the human-chimpanzee expression divergence estimates consistent across methodologies or showing large divergence in the sequencing data, but masked or undetected on the arrays (Table S6). Using rhesus macaque miRNA expression as an outgroup, 13 of the 25 could be assigned to the human evolutionary lineage and 8 to the chimpanzee evolutionary lineage (Table S6). Requiring significant support by at least two out of three methodologies (sequencing, microarrays and Q-PCR), expression changes in five miRNA (miR-184, miR-299-3p, miR-487a, miR-383 and miR-34c-5p) could be assigned to the human evolutionary lineage and two (miR-375 and miR-154*) to the chimpanzee evolutionary lineage (Figure 2). Six out of 7 miRNA assigned to the human- and the chimpanzee-evolutionary lineages in the prefrontal cortex also showed human- and chimpanzee-specific expression patterns in cerebellum (Figure 2).

### Effect of miRNA Expression Divergence on mRNA and Protein Expression

Do miRNA expression differences between human and chimpanzee brains contribute to gene expression divergence between these species? To estimate this, we measured mRNA and protein expression in human and chimpanzee prefrontal cortex: mRNA expression in five individuals of each species using Affymetrix Exon arrays, protein expression in four individuals of each species with two technical replicates using a label-free 2D-MS/MS Thermo-LTQ proteomics methodology (Table S1 and Table S7, Materials and Methods).

**Figure 3:**
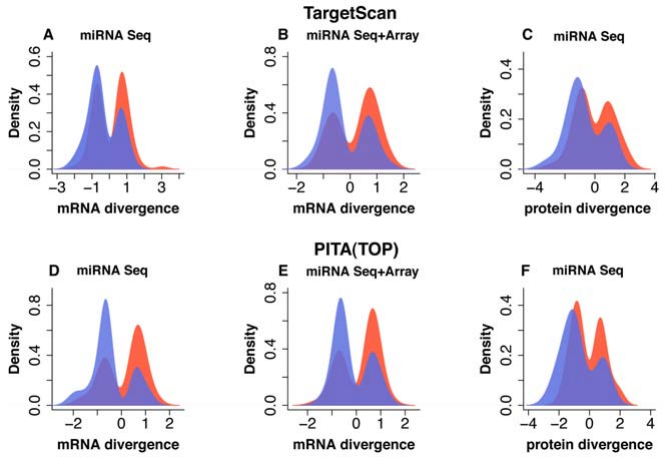
Effect of miRNA expression differences between humans and chimpanzees on mRNA and protein expression in prefrontal cortex. A-F, Distributions of expression divergence measurements (log2-transformed fold-change) for genes targeted by miRNA differently expressed between human and chimpanzee prefrontal cortex. Shown are: mRNA divergence distributions for 139 genes (A) and 106 genes (D), targeted by 37 miRNA classified as differently expressed based on high-throughput sequencing; mRNA divergence distributions for 97 genes (B) and 92 genes (E), targeted by 12 miRNA classified as differently expressed based on sequencing, as well as detected and showing consistent direction of expression difference on the microarrays; protein divergence distributions for 78 genes (C) and 64 genes (F) targeted by 37 miRNA classified as differently expressed based on high-throughput sequencing. Panels (A), (B) and (C) show target genes predicted using the TargetScan5 algorithm; panels (D), (E) and (F) - target genes predicted using PITA (TOP). The colors indicate genes targeted by miRNA that are: blue – miRNA highly expressed in human prefrontal cortex; red – miRNA highly expressed in chimpanzee prefrontal cortex. For both mRNA and protein divergence, positive values indicate higher gene expression in the human brain. Note that targets of highly expressed miRNA tend to show lower expression in the corresponding species. The purple areas show overlap between red and blue distributions. mRNA divergence is displayed as log2-transformed fold-change measurements between human and chimpanzee prefrontal cortex. Protein divergence is displayed as effect size measurements between human and chimpanzee prefrontal cortex.

Identified miRNA expression differences indeed had a significant negative effect on mRNA and protein expression in the human and chimpanzee prefrontal cortex, *i.e.*, targets of highly expressed miRNA were down-regulated in the corresponding species (Figure 3 and Table S8). This effect was significant for differentially expressed miRNA that were identified using both sequencing and microarray methodologies, as well as for miRNA that were identified by sequencing alone (Figure 3 and Table S8). The significance level of the effect did not depend on the choice of the miRNA target prediction algorithm: In brief, we obtained similar results using TargetScan5 predictions [10], [25] - based on the presence of conserved miRNA binding sites in mRNA 3′ UTR regions and reported to have good sensitivity and specificity [26] (Figure 3A-3C and Table S8) - as we obtained using PITA (TOP) predictions - based on the free energy gained from the formation of the miRNA-target duplex [27] (Figure 3D-3F and Table S8). Further, the negative effect of miRNA expression differences on mRNA and protein expression could be observed at various miRNA expression level cutoffs. For highly expressed miRNA, the negative effect on their targets' expression levels tended to be more significant (Table S8). Finally, the negative effect of miRNA on mRNA expression divergence between human and chimpanzee brains could also be reproduced at various mRNA expression divergence cutoffs (Figure S4E-S4F).

To assess an overall contribution of miRNA regulation to mRNA and protein expression divergence between human and chimpanzee brains, we calculated the proportion of significant mRNA and protein expression differences that could be negatively associated with miRNA expression differences. Since some of these associations might be caused by factors other than miRNA regulation, we used a number of significant mRNA and protein expression differences showing positive association between miRNA and target genes, as a background. At *p*<0.001 mRNA divergence cutoff (FDR<2%), 68 out of 479 (14%) mRNA, with significant expression differences between human and chimpanzee prefrontal cortex, could be negatively associated with miRNA expression differences. By contrast, 58 (12%) mRNA showed positive association. Thus, 2% of mRNA expression differences between human and chimpanzee brains could be assigned to miRNA regulation. Although this effect appears small, it can be observed consistently at all mRNA expression divergence cutoffs (Figure S4A-S4B). Further, at more stringent mRNA divergence cutoffs, the miRNA regulatory effect became more apparent reaching 4% at *p* = 0.0005. At the protein level, 26 out of 117 (22%) proteins with significant expression differences between humans and chimpanzees (FDR<5%) were negatively associated, and 21 (18%) - positively, with the miRNA expression divergence. Thus, we estimate that 4% of protein expression differences between human and chimpanzee brains could be caused by miRNA. Similarly, the miRNA regulatory effect could be consistently detected at all protein expression divergence cutoffs, and increased to 6% at *p* = 0.001 (Figure S4C-S4D).

These estimates are based on the assumptions that negative relationship between miRNA and target gene expression levels in the two species indicates regulation, while a positive relationship does not. Both assumptions might be incorrect. An excess of negative associations between miRNA and their predicted targets might be caused by yet unknown factors, rather than miRNA regulation. On the other hand, positive regulatory relationship between miRNA and target gene expression has been reported [28], [29]. Further, a positive correlation between miRNA and target gene expression could be caused by indirect regulatory effects [30]. Thus, the actual extent of the effect that miRNA have on gene expression divergence between adult human and chimpanzee brains remains to be estimated. Nevertheless, the consistent and significant negative relationship between miRNA expression, and the expression of their target genes, on both mRNA and protein levels (Figure 3 and Figure S4), and the consistent excess of negative associations between miRNA and their targets (Figure S4), demonstrates that miRNA expression divergence does contribute to gene expression divergence between humans and chimpanzees.

### Human-Specific miRNA Expression

To assess whether miRNA with expression divergence on the human lineage might be associated with human cognitive functions, we investigated the expression of genes targeted by five miRNA showing human-specific expression, according to multiple methodologies: miR-184, miR-487a, miR-383, miR-34c-5p and miR-299-3p (Figure 2). On the DNA sequence level, these miRNA tend to be conserved: miR-184 mature miRNA sequence is evolutionarily conserved from insects to humans, with only one nucleotide different at 3′end of mature sequence, while miR-383 and miR-34c-5p are classified as broadly conserved and miR-299-3p - as conserved among animal species [25], [31]. High sequence conservation indicates the functional importance of these miRNA and shows that expression divergence on the human evolutionary lineage is unlikely to be caused by lack of a selection constraint.

Notably, genes targeted by these five miRNA were enriched in neural functions. By contrast, no neural-related enrichment was observed for targets of the two miRNA showing chimpanzee-specific expression. Specifically, based on a functional analysis using DAVID [32], combined targets of the five miRNA with human-specific expression were significantly enriched in terms “signal transduction”, “synaptic transmission”, “cell surface receptor mediated signal transduction”, “neuronal activities” and “cell proliferation and differentiation” (Bonferroni-corrected *p*<0.05) (Table S9). Targets of miRNA with chimpanzee-specific expression were significantly enriched in terms “nucleoside, nucleotide and nucleic acid metabolism”, “mRNA transcription” and “mRNA transcription regulation” (Table S9).

Similarly, based on the DIANA-mirPath algorithm [33], targets of miR-184, miR-487a and miR-299-3p were significantly enriched in KEGG pathways that are related to neural functions (Table S10). Finding three out of five miRNA with significant target gene enrichment in neural functions was unexpected (permutation test, *p* = 0.067). Furthermore, miR-184 targets were significantly enriched in “long-term potentiation” pathway – one of the few pathways directly connected to learning and memory formation [34], [35]. Recent studies have also shown that miR-184 is involved in regulation of neural stem cell proliferation and differentiation [36]. Similarly, targets of miR-299-3p were significantly enriched in the “axon guidance” pathway, which is associated with neuronal cell differentiation and functions.

**Figure 4:**
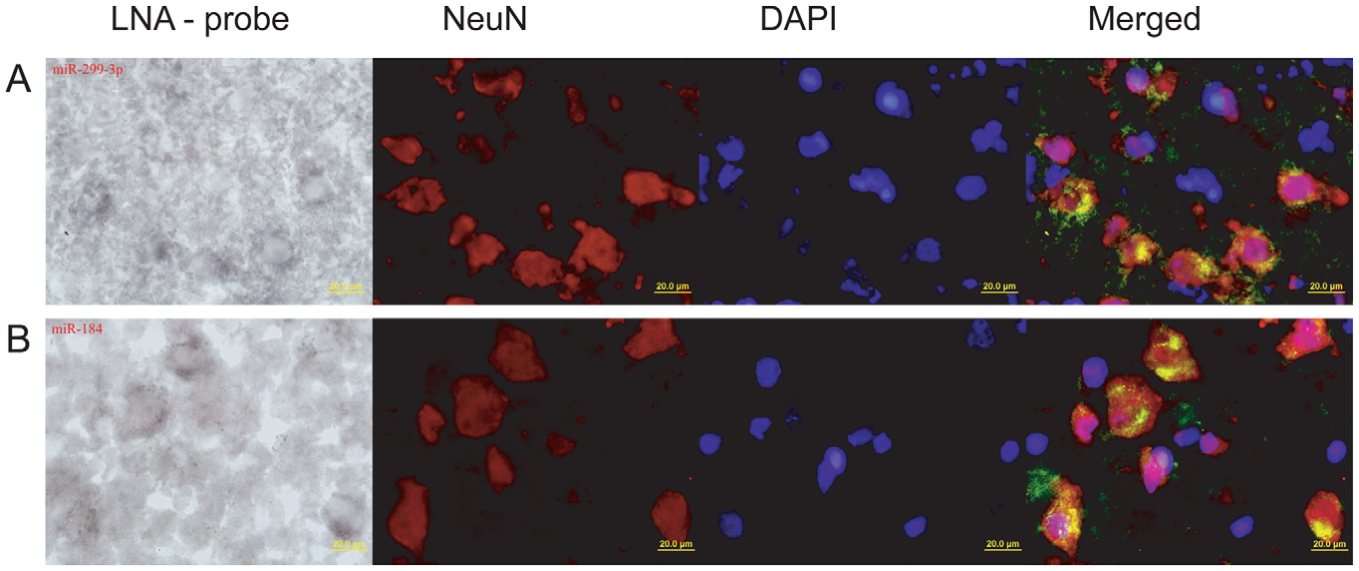
*In situ* staining of miR-184 and miR-299-3p in prefrontal cortex. (A) Rhesus macaque prefrontal cortex section hybridized with miR-299-3p LNA-probe (far left); anti-NeuN antibodies staining neuron nuclei (center left); DNA staining by DAPI (center right); and a merged image with miRNA staining shown in green (far right). (B) Human prefrontal cortex section hybridized with miR-184 LNA-probe (far left); anti-NeuN antibodies (center left); DAPI (center right); and a merged image (far right). All pictures were taken at 100x magnification. On the merged images, the miRNA hybridization signal was modified from its original one shown on the far left panel, by inverting and modifying to a green colour scale.

To further test association of miR-184 and miR-299-3p with neuronal functions, we determined their expression patterns in the human and macaque prefrontal cortex by *in situ* hybridization with specific LNA-probes (Table S11). Expression of the two miRNA co-localized with expression of NeuN protein, an established vertebrate neuronal-specific marker [37], [38] (Figure 4). Thus, even though pathway enrichment results were based on a limited number of miRNAs with species-specific expression in the prefrontal cortex, and although they relied on predicted miRNA-target relationships, both miRNA, with significant target gene enrichment in neuron-related pathways, are indeed preferentially expressed in neurons.

### Targets of miRNA with Human-Specific Expression

Due to the fact that miRNA functions cannot be tested in human or chimpanzee brains, we used two human neuroblastoma cell lines, SH-SY5Y and SK-N-SH, to verify miRNA target predictions and test their functions, as well as their expression changes on the human evolutionary linage. In order to achieve this, we transfected the two cell lines with double-stranded oligonucleotides, which mimic human mature miRNA sequences (Table S1, Materials and Methods). We tested the effects of all five miRNA showing human-specific expression in brain, as well as effects of the chimpanzee version of miR-299-3p sequence. The effects of each miRNA were assayed 24 hours after transfection using Affymetrix Human Genome U133 Plus 2.0 arrays. For each cell line, miRNA regulatory effects were calculated as the difference in expression levels between cells transfected with miRNA analogue and cells transfected with negative control oligonucleotides. For each cell line, transfection with negative control oligonucleotides was carried out in two independent replicates.

**Figure 5:**
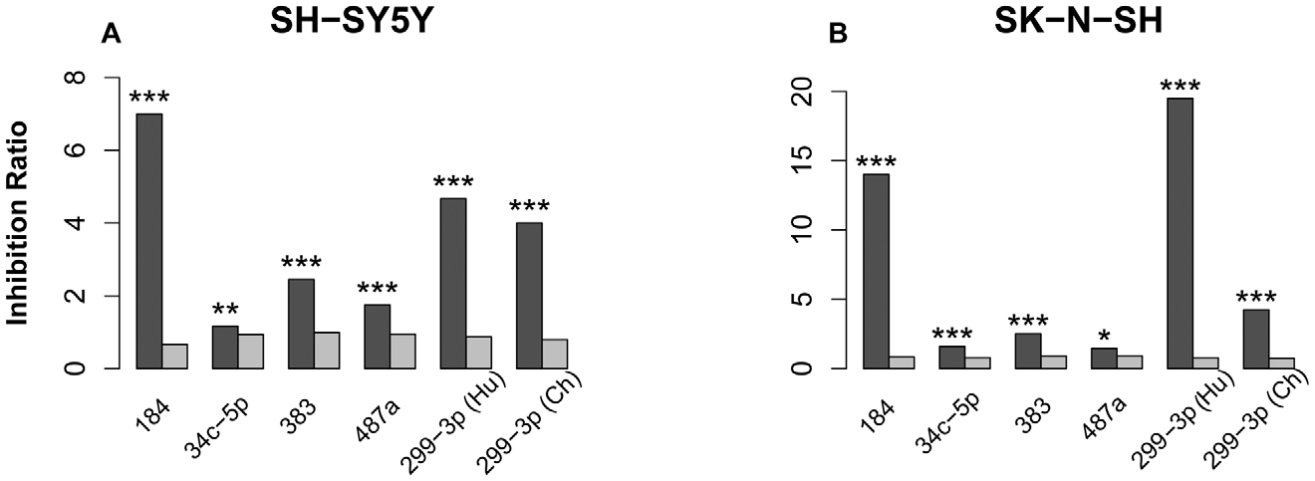
miRNA transfection effects in two cell lines. The dark grey bars depict the inhibition ratio of conserved miRNA targets, predicted using the TargetScan algorithm. The light grey bars depict the inhibition ratio of non-target genes. Inhibition ratio was calculated as the number of genes down-regulated 24 hours after miRNA transfection, divided by the number of not-down-regulated genes. For each gene, the miRNA transfection effect was calculated as a ratio of mRNA expression level 24 hours after miRNA transfection, divided by mRNA expression level 24 hours after transfection with negative controls (Materials and Methods). The significance of difference between target and non-target inhibition ratios estimated using Fisher's exact test is shown above the bars: *** - *p*<0.001; ** - *p*<0.01; * - *p*<0.05.

In both cell lines we observed significant expression inhibition of predicted miRNA targets for all 6 miRNA sequences (Figure 5). The results were highly consistent between two independent negative control replicates (Figure S5 and Figure S6) and showed significant overlap between the two cell lines (Figure S7). Further, in both cell lines we observed highly correlated target effects after transfection with human and chimpanzee versions of miR-299-3p. The mature sequence of miR-299-3p contains human-specific C to T substitution at position 10. While this substitution might affect relative stand selection efficiency during miRNA procession, as well as target selection, we did not find any significant differences in target effects between the human and the chimpanzee versions of miR-299-3p in our experiment (Figure S8).

To capture the majority of possible miRNA targets, we used 9 common target prediction algorithms. In agreement with a previous report [26], among the 9 algorithms, TargetScan resulted in better agreement between experimental results and target predictions (Table S12). It is noted, however, that targets predicted by other algorithms showed significant inhibition in transfection experiments. Thus, we used either TargetScan or we combined target predictions and down-regulation in cell line experiments to identify experimentally verified miRNA targets (Table S13 and Table S14, Materials and Methods).

Consistent with results based on computational predictions, the experimentally verified targets of miRNA with human-specific expression showed significant enrichment in certain neuronal functions. Specifically, based on a functional analysis using DAVID [32], experimentally verified targets of the five miRNA were significantly enriched, compared to genes expressed in the brain and in at least one of the two cell lines (Fisher's exact test, *p*<0.05), in the following biological processes and KEGG pathways associated with neural functions: “signal transduction”, “synaptic transmission”, “neurotransmitter release”, “adherens junction” and “axon guidance” (Tables S15).

**Figure 6:**
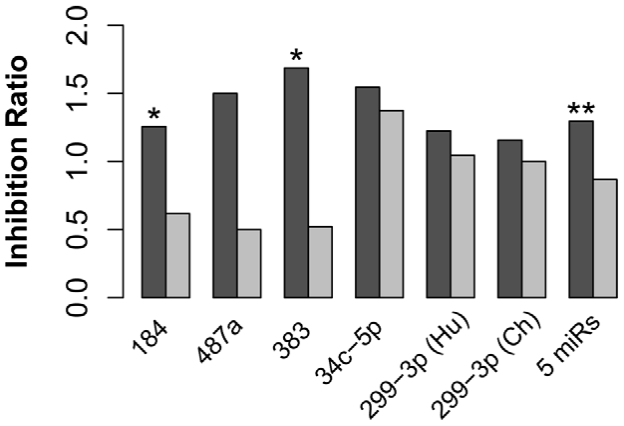
miRNA with human-specific expression showed negative association with expression of their target genes on the human evolutionary lineage. The dark grey bars depict the inhibition ratio of experimentally verified miRNA targets that showed human-specific expression on mRNA level in the prefrontal cortex. The light grey bars depict the inhibition ratio of the remaining targets of the same miRNA(s). Inhibition ratio was calculated as the number of genes showing opposite direction of expression divergence between human and chimpanzee brains, compared to that of the corresponding miRNA, divided by number of genes not showing this inverse expression divergence relationship. Experimentally verified miRNA targets were screened based on miRNA transfection experiments in two neuroblastoma cell lines (Materials and Methods). The significance of the inhibition ratio difference between miRNA targets with human-specific expression and miRNA targets with no human-specific expression were estimated using Fisher's exact test. The test significance is shown above the bars: *** - *p*<0.001; ** - *p*<0.01; * - *p*<0.05.

Notably, miRNA-target relationships experimentally verified in the two cell lines was also observed in brain. For each of the five human-specifically expressed miRNA we found inverse correlation between miRNA and target gene expression on the human evolutionary lineage (Figure 6). This effect was significant for combined targets of the five miRNA, as well as for targets of miR-184 and miR-383 analyzed individually. For the remaining miRNA regulatory effects were not significant, but they did show target expression inhibition. Thus, miRNA-target relationship identified in cell line experiments did allow us to capture miRNA-target relationship, thus explaining some of gene expression changes that took place in the brain on the human evolutionary lineage.

### Timing of miRNA Expression Divergence

While human and chimpanzee evolutionary lineages separated approximately 6–7 million years ago, humans and Neanderthals shared a common ancestor less than half a million years ago [39]. Thus, using Neanderthal data it might be possible to date miRNA expression change more precisely. Although miRNA expression in Neanderthal brain cannot be estimated, signature of positive selection spanning miRNA promoter, or the regulatory region in the human genome, would indicate that expression change might have taken place after human and Neanderthal linage separation [40].

**Figure 7:**
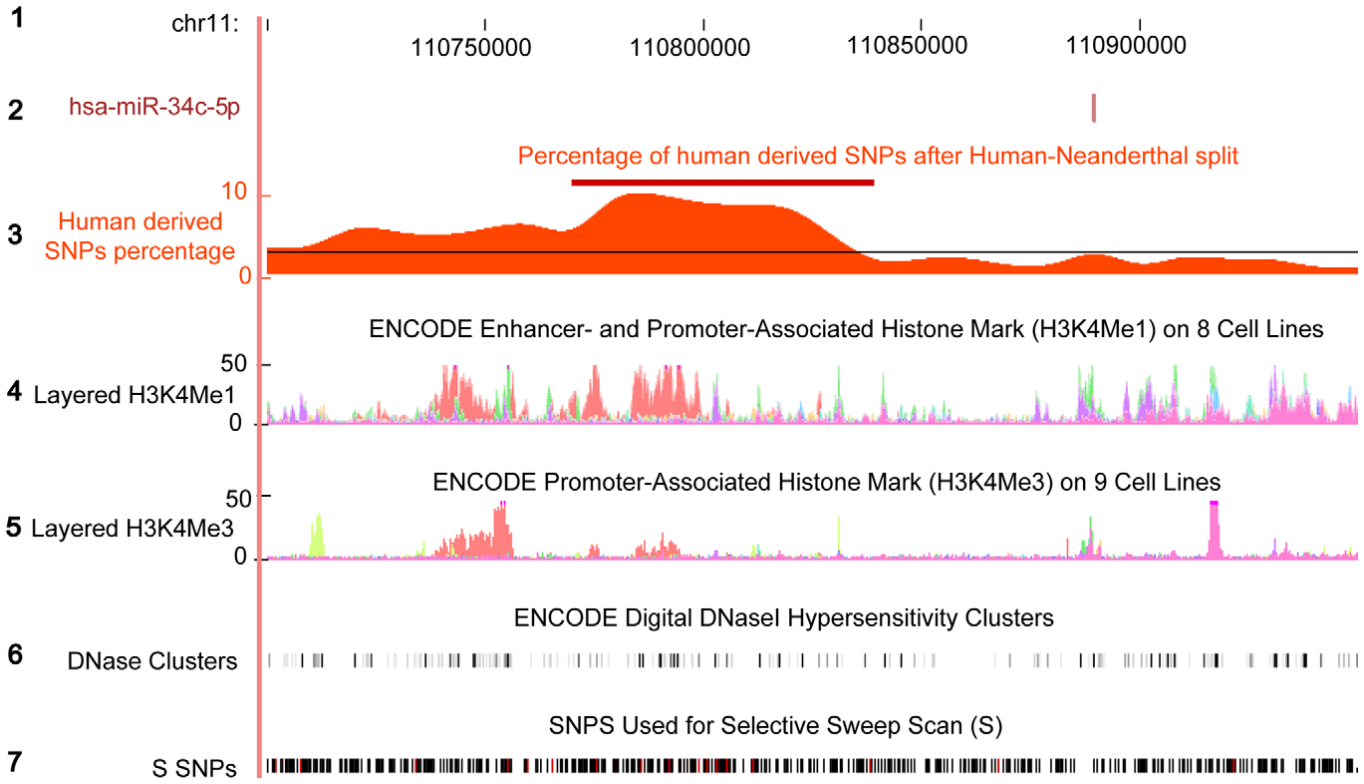
Excess of human derived SNPs in the upstream region of hsa-miR-34c. The plot shows 150kb region upstream of human miR-34c. The region annotation, from top to bottom: (1) The human genome coordinates based on hg18; (2) The genomic location of miR-34c-5p miRNA; (3) The percentage of human derived SNPs out of all SNPs calculated within 50kb sliding windows. The red bar shows the sliding windows with the significant excess of human derived SNPs, compared to the genome average (Fisher's exact test, Bonferroni corrected *p*<0.05). The average genome percentage is depicted by the black line (Materials and Methods); (4) H3K4Me1 histone modification density in eight cell lines from ENCODE database [66]. Presence of this histone mark indicates enhancer and, to a lesser extent, promoter activity [67]; (5) H3K4Me3 histone modification density in nine cell lines from ENCODE database [66]. Presence of this histone mark is associated with promoters [67]. Note that the genomic region showing the highest density of human derived SNPs overlaps with enhancer activity, but not with the promoter histone modification mark; (6) DNase Hypersensitivity Clusters from ENCODE database [66]. Regulatory regions tend to have higher DNase sensitivity [68]; (7) SNPs used in a genome-wide scan for signals of positive selection in the human lineage since divergence from the Neanderthal lineage [40]. Human derived SNPs are shown in red.

We indeed found a significant excess of human derived SNPs, indicating the presence of positive selection on the human evolution linage after the human-Neanderthal split, in the upstream regions of one out of five miRNA with human-specific gene expression: miR-34c-5p (Fisher's exact test, Bonferroni corrected *p*<0.05, Materials and Methods). Genome-wide, the possibility of finding a signature of positive selection at this significance level within the upstream region of five randomly chosen miRNA is low (1000 permutations, *p*<0.05). Notably, for miR-34c-5p signature of positive selection was located in the putative enhancer region approximately 100kb upstream of the miRNA gene (Figure 7). Thus, although indirectly, these results indicate that the change in miR-34c-5p with human-specific expression might have taken place after the separation of the human and the Neanderthal evolutionary lineages. Furthermore, positive selection on changes in regulatory regions of this miRNA indicates their potential adaptive significance.

Functionally, miR-34c-5p was previously shown to be down-regulated in cancer and Parkinson disease [41]–[44]. We further characterized possible functions of miR-34c-5p in the human brain, based on target genes experimentally verified in cell lines. Compared to the genes expressed in brain, these target genes were significantly enriched, among others, in biological processes “neurotransmitter secretion” and “behaviour”, as well as cellular components “dendrite cytoplasm”, “synapse” and “cell junction” (Fisher's exact test *p*<0.01, Tables S16). These findings indicate that changes in miR-34c-5p expression on the human evolutionary linage might have resulted in gene expression changes affecting cognitive functions.

In conclusion, despite high sequence conservation of 325 miRNA expressed in the prefrontal cortex, 11% were expressed at significantly different levels in humans and chimpanzees. The vast majority of these differences were also found in cerebellum and were confirmed by microarray and Q-PCR experiments. Importantly, we observed significant inverse relationship between human-chimpanzee miRNA expression divergence and expression divergence of the predicted target genes at both mRNA and protein levels. This indicates that miRNA expression divergence plays an important role in shaping gene expression divergence among species.

Approximately half of the miRNA expression differences found in the prefrontal cortex could be assigned to the human evolutionary lineage. These miRNA, as well as their target genes, were conserved at the sequence level. Thus, their expression divergence is unlikely to be explained by a lack of selective constraints. Instead, targets of miRNA with human-specific expression were enriched in neural functions associated with learning and memory pathways, such as “axon guidance” and “long term potentiation”. Potential influence of miRNA divergence on neuronal functions was further confirmed by preferential expression of the corresponding miR-299-3p and miR-184 in cortical neurons, as well as verification of the predicted miRNA-target relationship in two human neuroblastoma cell lines. Based on miRNA-target relationships verified in cell lines, we further demonstrated the effect of miRNA regulation on gene expression changes in brain, on the human evolutionary lineage. Finally, we show that at least one out of five human-specific miRNA expression changes found in brain might have occurred after separation of the human and the Neanderthal evolutionary lineages. Signature of positive selection found in the enhancer region of the miRNA, miR-34c-5p, further indicates that this change might have had adaptive significance.

Although these findings do not provide direct evidence that miRNA regulation resulted in human-specific phenotypic adaptations, taken together they indicate that miRNA regulation did contribute to gene expression changes on the human evolutionary lineage and that it affected genes involved in neuronal functions. Further studies are needed to evaluate functional significance of the miRNA-driven transcriptome changes.

## Materials and Methods

### Ethics Statement

Informed consent for the use of human tissues for research was obtained in writing from all donors or their next of kin. All non-human primates used in this study suffered sudden deaths for reasons other than their participation in this study and without any relation to the tissue used. Biomedical Research Ethics Committee of Shanghai Institutes for Biological Sciences completed the review of the use and care of the animals in the research project (approval ID: ER-SIBS-260802P).

### Illumina Sequencing Experiment

Human tissue was obtained from the NICHD Brain and Tissue Bank for Developmental Disorders at the University of Maryland, Baltimore, MD. The role of the NICHD Brain and Tissue Bank is to distribute tissue and, therefore, cannot endorse the studies performed or the interpretation of results. All subjects were defined as normal controls by forensic pathologists at the NICHD Brain and Tissue Bank. No subjects who suffered a prolonged agonal state were used. For the prefrontal cortex, samples were taken from the frontal part of the superior frontal gyrus: a cortical region approximately corresponding to Brodmann Area 9. For all samples, similar proportions of grey and white matter were dissected. Total RNA was isolated from the frozen prefrontal cortex tissue using the Trizol (Invitrogen, USA) protocol with no modifications. Prior to low molecular weight RNA isolation, the total RNA from 20 male individuals aged between 14 and 58 years was combined in equal amounts. Low molecular weight RNA was isolated, ligated to the adapters, amplified and sequenced following the Small RNA Preparation Protocol (Illumina, USA) with no modifications. Technical replication was completed by independently processing the mixed sample of 20 individuals starting from the low molecular weight RNA isolation step. We carried out the sample preparation and deep sequencing by choosing 5 adult chimpanzee individuals and 5 rhesus macaque individuals following the protocols used for human samples. Details of all samples are given in Table S1. All original deep sequencing data is deposited in the NCBI GEO database [GSE26545].

### Agilent miRNA Microarray Experiment

Total RNA was isolated using the mirVana miRNA isolation kit (Ambion). 100 ng of each RNA sample were hybridized to Agilent Human microRNA Microarray (G4471A, Agilent Technologies). MicroRNA labelling, hybridization and washing were carried out following Agilent's instructions [45].

Agilent microRNA assays integrate eight individual microarrays on a single glass slide. Each microarray includes approximately 15 k features containing probes sourced from the miRBase public database. The probes are 60-mer oligonucleotides directly synthesized on the array. We used Human miRNA Microarray Version3, which contains probes for 866 human and 89 human viral microRNAs from the Sanger miRBase v12.0. All samples used in the prefrontal cortex comparison among three species were hybridized to one array. Data from samples hybridized on a single array were processed and analyzed separately to avoid possible batch effects.

Images of hybridized microarrays were acquired with a DNA microarray scanner (Agilent G2565BA); Feature Extraction software v.10.5.1.1 (Agilent G4462AA) was uses for image analysis with default protocols and settings.

As miRNA microarray probes are based on human mature miRNA sequences, expression levels of miRNA with sequence differences among species cannot be measured reliably. All probes corresponding to 150 such miRNA between human and chimpanzee and 313 such miRNAs between human and rhesus macaque present on the array were masked prior to expression level analysis, based on the mature sequence comparison.

### Affymetrix Exon Array Experiment

mRNA samples for Affymetrix Human Exon 1.0 ST Arrays were prepared following the standard GeneChip Whole Transcript (WT) Sense Target Labelling Assay. We processed Exon Array datasets following the steps described in [46]. We processed the human, chimpanzee and rhesus macaque datasets separately. For the human dataset, in order to identify array probes that contain mismatches and multiple locations to human genome (hg18), we mapped Human Exon 1.0 ST probes to the human genome using Bowtie [47]. Based on these alignments, we included probes that matched the genome perfectly and at a single location. For the rhesus macaque and chimpanzee datasets, we applied the same procedure by mapping probes to the rhesus macaque genome (MMUL1.0) and the chimpanzee genome (panTro2.1) separately. Finally, we chose probes that match the (i) human and chimpanzee genomes for human and chimpanzee gene expression comparison and, (ii) all three species' genomes for human, chimpanzee and rhesus macaque gene expression comparison. To determine whether the signal intensity of a given probe was above the expected level of background noise, we compared the signal intensity for each probe to a distribution of signal intensities of the anti-genomic probes with the same GC content. Anti-genomic probes are specifically designed by Affymetrix to provide an estimate of the non-specific background hybridization [48]. A probe was classified as detected if its intensity was larger than the 95% percentile of the background probes with the same GC content [48]. To further remove any possible systematic experimental bias among arrays, we performed a PM-GCBG correction and quantile normalization using the R package "preprocessCore" (http://svitsrv25.epfl.ch/Rdoc/library/preprocessCore/html/00Index.html). Prior to norm-alization, all intensities were log2 transformed. A transcript was classified as detected if more than 80% of probes and at least ten probes per transcript were classified as detected. The intensities of transcripts were summarized by the median polish method. We used the Transcript Cluster Annotations file to map the transcript clusters annotated by Affymetrix to Ensembl genes (Ensembl54). In cases where multiple transcript clusters mapped to the same gene, we calculated gene expression as the median of all corresponding transcript clusters. None of the transcript clusters overlapped. All original microarray data is deposited in the NCBI GEO database [GSE26545].

### Sample Preparation and Label-Free 2D-MS/MS Thermo-LTQ Proteomics Methodology

Protein sample preparation and 2D LC-MS/MS analysis and peptide identification are described elsewhere [46]. Briefly, proteins were extracted from 100 mg of frozen cerebellar tissue samples. The resulting protein solution was incubatedovernight with Trypsin, followed by ultrafiltration and lyophilization. Lyophilized protein samples werethen dissolved in a loading buffer for the LC-MS/MS analysis. Peptide fractionation and analysis were performed in a pH continuous online gradient (pCOG) 2D LC-MS/MS system. Peptide identification was achieved by searching against a database of human peptides (IPI human v3.22) and its reversed version representing mock database using SEQUEST program in BioWorks 3.2 software suite. A mass tolerance of 3.0 Da and one missed cleavage site of trypsin were allowed. Cysteine carboxyamidomethlation was set as static modification and no other modification was checked. All output results were filtered and integrated to proteins by an in-house software “BuildSummary”. Using a false discovery rate (FDR) of less than 0.5%, all of the matches passing a certain Xcorr and delta CN were regarded as valid. Further, all the peptides that could be assigned to multiple proteins were removed. All identified protein IDs were mapped to Ensembl gene IDs using Biomart. Protein expression of each gene was calculated as a median copy number of all peptides, assigned uniquely to any of the isoforms of the corresponding gene. Genes with more than 5 peptides identified in human and chimpanzee brains were used in the miRNA target effect analysis. Based on this cutoff, we quantified protein expression for a total of 981 genes. The processed protein dataset is provided in Table S7.

### Quantitative RT–PCR

For mature miRNA quantification we used the TaqMan MicroRNA Assay (Applied Biosystems) system [49]. cDNA was synthesized from 50 ng total RNA from in a 15 µl reaction volume, according to the TaqMan MicroRNA Assay protocol. By using hairpin primers targeting specifically mature miRNAs, reverse transcription was performed using the following program: 30 min at 16°C, 30 min at 42°C, 5 min at 85°C and then held at 4°C. For relative quantification by real time, 1.5 µl cDNA were used in a total reaction volume of 20 µl with 1 µl custom TaqMan assay using a Roche LC480 RT PCR System. Each measurement was performed in triplicate for each assay. At least two biological replicates for each species were used. Ct (threshold cycle) values of RT PCR were normalized to the endogenous control U6 measured together with the test samples. The relative expression of each miRNA was calculated as log2 of 2-Ct values.

### Mapping of Sequence Reads to the Human, Chimpanzee, and Rhesus Macaque Genomes

We mapped the deep sequencing data following the mapping steps of [50]. For each of the brain sequencing datasets, to remove the adapter sequence at the 3′-end of the sequence reads, all unique sequences were trimmed using the custom trimming procedure. The trimmed sequences of each species were mapped to the corresponding genomes, human genome (hg18), chimpanzee (PanTro2.1) and rhesus macaque (MMUL1.0), using SOAP2 algorithm [51]. Only sequences perfectly matching the genome and with a length ranging from 18 to 28 nucleotides were retained.

### Known and Novel Star miRNA Quantification

We quantified the miRNAs expression following the quantification steps of [50]. First, all sequences with at least one read mapping within three nucleotides upstream or downstream of the 5′-position of the mature miRNAs were retained. Then, for each mature miRNA, the sequence with a maximal copy number was designated as the reference sequence. Finally, the expression level of each miRNA was calculated as the sum of the copy number of the reference sequence and the sequences mapping at the same 5′-end position as the reference sequence. Besides the quantification of known miRNAs, novel miRNAs were detected following [50]. Specifically, for the miRNA precursors with one annotated miRNA, small sequences mapping to the opposite arm of the precursor hairpin were analysed. The sequence with the maximal copy number was considered as a novel miRNA candidate. A further criterion required the existence of at least 14 basepairs between an annotated miRNA and a novel miRNA candidate within the precursor hairpin. The quantification process for novel miRNAs was the same as for known miRNAs.

### miRNA Ortholog Finding in the Chimpanzee and Rhesus Macaque Genomes

Human microRNA information was downloaded from miRBase version 12 [52]-[54]. We used two steps for the ortholog finding; first, we extracted the best precursor orthologs by using a combination of reciprocal BLAT, BLAST and liftOver in chimpanzee and rhesus macaque genomes. Specifically, we mapped all annotated human miRNA precursors to the chimpanzee and rhesus macaque genomes using reciprocal BLAT, BLAST and liftOver, and required one precursor ortholog to be supported by at least 2 out of 3 methods. For reciprocal BLAT, we chose the following parameter configuration: [-stepSize = 5 -repMatch = 2253 -minScore = 0 -minIdentity = 0]. We further required the length of each hit sequence to be more than 70% and less than 130% of the query sequence. For reciprocal BLAST, we chose the parameter configuration [-F F -b 1 –e 10^−5^] and again required the length of hit sequence to be more than 70% and less than 130% of query sequence. For reciprocal liftOver, we chose the website parameter configuration with Perl LWP module [hglft_minMatch = >0.6, hglft_minSizeT = >0, hglft_minSizeQ = >0 boolshad.hglft_multiple = >0] and similarly required the length of the hit sequence to be more than 70% and less than 130% of query sequence.

We next extracted mature miRNAs based on aligned precursor sequences using ClustalW2 and Muscle, with default parameters. The extracted mature sequence by ClustalW2 and Muscle were highly consistent (<0.1% difference).

### miRNA Differential Expression Detection

The procedure for identifying differentially expressed miRNAs in deep sequencing data was as follows: We normalized data from two species belonging to the same brain region (*e.g.* human and chimpanzee prefrontal cortex) using quantile normalization. We then used statistical significance, fold-change and detection level as criteria for differential expression (Fisher's exact test *p*<0.01, fold-change>2, at least 10 sequence reads in at least one of the two species). We further required that the candidate miRNA should fulfil these criteria in both technical replicates in the prefrontal cortex. Normalization by the number of the total mapped reads (transcripts per million, TPM) produced almost identical results [data not shown].

Alternatively, to identify miRNA differentially expressed between humans and chimpanzees or between humans and macaques, we applied a procedure implemented in the edgeR package [23] using the following criteria: *p*<0.001, FDR<0.01.

For identifying differentially expressed miRNA in Agilent miRNA microarray data, a similar approach was used. We first quantile normalized data contained within one Agilent array, and then used both statistical significance and fold-change as criteria for differential expression (Student t-test, *p*<0.01, fold-change>2).

### miRNA Target Effects' Detection

For the miRNA differently expressed between humans and chimpanzees, we expected targets of miRNA highly expressed in humans to be down-regulated in humans. We first used TargetScan5 [10], [25] to predict the miRNA targets as this algorithm is reported to have relatively high sensitivity and specificity [26]. To test target effects on the mRNA level, we normalized gene expression between species using quantile normalization and excluded genes with absolute difference between species smaller than 0.5. Using the Wilcoxon signed-rank test, we then compared the expression difference between the targets of miRNA that were highly expressed in humans with targets of miRNA that were highly expressed in chimpanzees. Before applying the Wilcoxon signed-rank test, the genes that were targeted by both miRNA highly expressed in humans and miRNA highly expressed in chimpanzee (*i.e.* targets with inconsistent miRNA effects) were excluded.

Due to greater intra-species variation in the protein data, when testing the miRNA target effects on protein expression, we revised the method to use the effect size to represent the expression difference between species. Only genes with absolute effect size greater than one were used in analysis.

To check the robustness of detected target effect at both mRNA and protein levels, we used different expression level cutoffs for identification of differentially expressed miRNA, which yielded qualitatively the same result as reported in the main text (Table S8). We further determined that the target effects could be reproduced using another target prediction algorithm, PITA (TOP) [27].

To calculate the percentage of differently expressed genes that could be explained by miRNA expression divergence between human and chimpanzee, we first identified differentially expressed genes with FDR less than 2% at the mRNA level and less than 5% at the protein level (FDR was estimated using 1000 permutations). Among these genes, we determined the percentage that were targeted by differentially expressed miRNA, where at least one miRNA-target gene pair showed expression change in opposite directions.

### miRNA Transfection and Microarray Experiments

miRNA transfection experiments were conducted on two human derived neuroblastoma cell lines (SH-SY5Y and SK-N-SH) (Table S1). Briefly, cells were plated in 0.5 ml of growth medium, without antibiotics, 24 h prior to transfection.miRNA mimics-Lipofectamine 2000 (Invitrogen) complexes were prepared freshly before transfection according to the manufacturer's protocol.SH-SY5Y and SK-N-SH cells were transfected in six-well plates using miRNA mimics-Lipofectamine 2000 with a final oligonucleotide concentration of 10 nmol/L. In parallel, negative control transfections with mock oligonucleotides were conducted according to the manufacturer's protocol. For each cell line, transfections with negative control oligonucleotides were carried out in two independent replicates. Cells were harvested after 24 h, total RNA were extracted with Trizol reagent(Invitrogen) and further processed and hybridized to Affymetrix Human Genome U133 Plus 2.0 arrays following the manufacturer's instructions. The gene expression levels were determined using R *RMA* package. All original microarray data are deposited in the NCBI GEO database [GSE26545].

### Functional Analysis of miRNA with Species-Specific Expression

We used DIANA-mirPath [33] to determine putative functions of species-specific miRNA. DIANA-mirPath is a web-based computational tool that has been developed to identify molecular pathways potentially altered by the expression of single or multiple microRNAs [33]. The software performs an enrichment analysis of multiple microRNA target genes by comparing each set of microRNA targets to all known KEGG pathways. We chose TargetScan5 and PicTar as target prediction tools and required a score threshold of 6.9 (*p*<0.001) (Table S10). Based on the DIANA-mirPath algorithm, targets of miR-184, miR-487a and miR-299-3p were significantly enriched in KEGG pathways that are related to neural functions (Table S10). To test global significance of this result, 1000 simulations were done by randomly choosing five miRNA out of all 325 human miRNA expressed in brain (Table S3) and applying the same test procedure. In 67 out of 1000 simulations, we observed three or more miRNA with enriched KEGG pathways that related to neural functions equal to or larger than the ones observed in the real data (permutations, *p* = 0.067).

In a parallel approach, the DAVID tool for functional annotation of gene sets [32] was used to investigate the putative functions of genes targeted by human-specific or by chimpanzee-specific miRNAs, as predicted by TargetScan. Genes expressed in brain and targeted by human annotated miRNA were taken as background. Significant enriched biological processes based on the PANTHER (Protein ANalysis THrough Evolutionary Relationships) Classification System are listed in Table S9 (Benjamini-Hochberg corrected Fisher's exact test *p*<0.05) [55].

Further, we used DAVID to investigate putative functions of experimentally verified target genes of miRNAs with human specific expression, based on our transfection results. Experimentally verified target genes were predicted by TargetScan and were required to show down-regulation by transfection of the corresponding miRNA in at least one of the two cell lines (Table S13). Experimentally verified target genes expressed in brain were used in functional enrichment analyses. Genes expressed in both brain and at least one of two cell lines were used as a background. Significantly enriched biological processes based on the PANTHER Classification System and KEGG pathways are listed in Table S15 (Fisher's exact test *p*<0.05).

### Effect of miRNA Regulation on Human-Specific Gene Expression Changing in Brain

To capture the majority of possible miRNA targets, including non-conserved ones, we combined predictions of 9 algorithms: TargetScan5 [10], PITA [27], PicTar [56], mirSVR [57], MirTarget2 [58], microT v3.0 [59], TargetMiner [60], Antar [61] and 2step-SVM [62] (Table S12). In order to classify predicted targets as experimentally verified, we calculated target FDR, for each algorithm, based on the inhibitory effect observed in cell line transfection experiments. Specifically, we calculated proportions of predicted target genes and non-target genes inhibited after transfection in both cell lines, at a certain inhibition cutoff (calculated as the difference in expression between miRNA transfection and the negative control). FDR was calculated as the ratio of the proportion of non-target genes passing this inhibition cutoff compared to the total proportion of target genes expressed in the corresponding cell line. The unions of targets predicted by the 9 algorithms at FDR<10% cutoff were used as experimental verified miRNA targets, except for miR-34c-5p, which target FDR was taken at 15% due to a weaker inhibition effect, observed in our transfection experiment (Table S14).

Genes with human-specific and chimpanzee-specific expression were determined by comparison of human-macaque and chimpanzee-macaque expression distances. Genes with greater human-macaque distance were classified to have human-specific expression. Although this requirement is non-conservative, it results in enrichment for genes with human-specific expression. Further, strict identification of human-specific gene expression changes was not a focus of this study. Fisher's exact test was used to determine whether genes with human-specific expression, and showing inverse expression change compared to a given miRNA, were enriched among experimental verified targets of this miRNA. Target genes of this miRNA that were not showing human-specific expression were used as a background.

### In Situ Hybridizations

We designed two LNA-probes complementary to miR-184 and miR-299-3p respectively (Table S11). Hybridizations were performed as described in [63]. Briefly, 10 micrometer-thick tissue sections were collected on Superfrost/plus slides (Fisher). After washing in two changes of excess PBS, sections were acetylated with 0.1M triethanolamine/0.25% acetic anhydride for 10 minutes and then incubated in humidified bioassay trays for prehybridization at 55°C (20–25°C below the Tm of the probe) for 4 hours in hybridization buffer (5xSSC/lx Denhardt's solution/5 mM EDTA/0.1% Tween/0.1% DHAPS/50% deionized formamide/0.1 mg/ml Heparin and 0.3 mg/ml yeast tRNA) [63], [64]. This procedure was followed by an over-night hybridization step using a DIG-labelled LNA oligonucleotide probe complementary to the target miRNA. Below the temperature of 55°C, sections were rinsed and washed twice in 2xSSC and 3 times in 0.2xSSC. The *in situ* hybridization signal was detected by incubation with alkaline phosphatase (AP)-conjugated anti-DIG antibody, using NBT/BCIP as substrate for 3–12 minutes.

### Positive Selection in miRNA Regulatory Regions

SNPs used in a genome-wide scan for signals of positive selection in the human lineage, since divergence from the Neanderthal lineage (Selective Sweep Scan or S SNPs) [40], were downloaded from UCSC. Following the published procedure, SNPs were defined as human derived when at least four out of six modern human genomes were derived while all observed Neanderthal alleles were ancestral [40]. An overrepresentation of human derived SNPs in a region would imply that the region had undergone positive selection in the modern human lineage, since divergence from Neanderthals. 50kb sliding windows with a 10 kb step were used to scan the human derived SNPs along the human genome. For five human specific miRNA, we used Fisher's exact test to check overrepresentation of human derived SNPs in each sliding window, with 150 kb region upstream of the annotated miRNA precursor. Four windows in an upstream region of miR-34c-5p were significant at Bonferroni corrected *p*<0.05. To test the global significance of this result, 1000 simulations were performed by randomly choosing five miRNA precursors out of all 622 annotated human miRNA precursors, and the same test procedure applied. In 44 out of 1000 simulations we observed four or more sliding windows with Fisher's exact test p-values equal to, or smaller than, the ones observed in the real data (permutation *p* = 0.044). Putative functions of miR-34c-5p targets were determined using CORNA [65], using experimentally verified target genes of miR-34c-5p as predicted by the 9 aforementioned algorithms, at FDR = 15%. Genes expressed in brain were used as a background.

## Supporting Information

Figure S1 Correlations between miRNA expression profiles. The Pearson coefficients of miRNA expression level correlations between and within species based on 572 miRNA detected in the prefrontal cortex samples of all three species in small-RNA-seq data. The labels represent species: Hu – human; Ch – chimpanzee; Ma – rhesus macaque.
(TIF)

Figure S2 miRNA divergence between species measured in the prefrontal cortex and cerebellum. miRNA expression divergence (log2-transformed fold-changes) between humans and chimpanzees (*N* = 37) (A and B) or humans and rhesus macaques (*N* = 106) (C and D) measured using high-throughput sequencing in prefrontal cortex and cerebellum. The black dots indicate miRNA showing consistent direction of expression divergence in the two brain regions; grey dots – miRNA showing inconsistent directions of expression changes. The labels represent species: Hu – human; Ch – chimpanzee; Ma – rhesus macaque. Hu1 and Hu2 are two biological replicates of the human cerebellum sample.
(TIF)

Figure S3 Q-PCR validation of species-specific miRNA expression. The histogram shows normalized miRNA expression levels in human (dark grey) and chimpanzee (light grey) prefrontal cortex samples measured using Q-PCR. The y-axis shows average miRNA expression normalized to the expression of invariant transcript (U6 snRNA). The error bars show one standard deviation based on duplicate Q-PCR experiments in three human and three chimpanzee samples. The symbols indicate significance levels: Student's t-test *p*<0.05 - *; *p*<0.01 - **; *p*<0.001 - ***.
(TIF)

Figure S4 miRNA effect on mRNA and protein expression differences between human and chimpanzee prefrontal cortex (PFC). (A) Percentage of target mRNAs negatively (red curve) and positively (black curve) associated with 37 miRNAs differentially expressed in human and chimpanzee PFC at different mRNA divergence cutoffs. The mRNA divergence cutoff is based on Student's t-test p-values calculated using gene expression in human and chimpanzee individuals. (B) Numbers of target mRNA negatively (red bars) and positively (black bars) associated with 37 miRNAs differentially expressed in human and chimpanzee PFC at different mRNA divergence cutoffs shown in panel A. (C) Percentage of target proteins negatively (red curve) and positively (black curve) associated with 37 miRNAs differentially expressed in human and chimpanzee PFC at different protein divergence cutoffs. The protein divergence cutoff is based on Student's t-test p-values calculated using protein expression in human and chimpanzee individuals. (D) Numbers of target proteins negatively (red bars) and positively (black bars) associated with 37 miRNA differentially expressed in human and chimpanzee PFC at different protein divergence cutoffs shown in panel C. (E) Difference in median expression divergence between mRNA targeted by miRNA with high expression in the human PFC (blue curve), and mRNA targeted by miRNA with low expression in the human PFC (red curve), at different mRNA divergence cutoffs. Difference in median expression divergence between two mRNA groups was significant at each of the mRNA divergence cutoffs (Wilcoxon signed rank text, p<0.05). mRNA divergence cutoff was calculated based on absolute mean difference between human and chimpanzee expression levels. (F) Numbers of target genes for each mRNA divergence cutoff shown in panel E.
(TIF)

Figure S5 Target effect in two independent negative control replicates (Mock 1 and Mock 2) in SH-SY5Y cell line. The red points represent the target effect of conserved targets, predicted by TargetScan, with corresponding miRNA. The black points represent the background expression – all expressed genes, excluding targets of the miRNA, are shown. Target effect (miRNA regulatory effect) was calculated as the log2-transformed expression level difference between cells transfected with miRNA analogue and cells transfected with negative control (mock) oligonucleotides. The red legend in the bottom right corner of each plot shows the Pearson correlation coefficient of target effect using two negative controls (Mock 1 and Mock 2) in SH-SY5Y cell line (R) and correlation p-value (p). The X-axis shows the target effect using Mock 1 as a negative control, the Y-axis shows target effect using Mock 2 as a negative control.
(TIF)

Figure S6 Target effect in two independent negative control replicates (Mock 1 and Mock 2) in SK-N-SH cell line. The red points represent the target effect of conserved targets, predicted by TargetScan, with corresponding miRNA. The black points represent the background expression – all expressed genes, excluding targets of the miRNA, are shown. Target effect (miRNA regulatory effect) was calculated as the log2-transformed expression level difference between cells transfected with miRNA analogue and cells transfected with negative control (mock) oligonucleotides. The red legend in the bottom right corner of each plot shows the Pearson correlation coefficient of target effect using two negative controls (Mock 1 and Mock 2) in SK-N-SH cell line (R) and correlation p-value (p). The X-axis shows the target effect using Mock 1 as a negative control, the Y-axis shows target effect using Mock 2 as a negative control.
(TIF)

Figure S7 Target effect of miRNA transfection in two neuroblastoma cell lines. The red points represent the target effect of conserved targets, predicted by TargetScan, with corresponding miRNA. The black points represent the background expression – all expressed genes, excluding targets of the miRNA, are shown. Target effect (miRNA regulatory effect) was calculated as the log2-transformed expression level difference between cells transfected with miRNA analogue and cells transfected with negative control (mock) oligonucleotides. The red legend in the bottom right corner of each plot shows the Pearson correlation coefficient of the target effect between two cell lines, SH-SY5Y and SK-N-SH, calculated using Mock 1 as a negative control (R) and the correlation p-value (p). The X-axis shows target effect in SH-SY5Y cell line, the Y-axis shows target effect in SK-N-SH cell line.
(TIF)

Figure S8 Target effects of human and chimpanzee variants of miRNA 299-3p (hsa-miR-299-3p and ptr-miR-299-3p) after transfection in two cell lines. The red points represent the target effect of conserved targets, predicted by TargetScan, with corresponding miRNA. The black points represent the background expression – all expressed genes, excluding targets of the miRNA, are shown. Target effect (miRNA regulatory effect) was calculated as the log2-transformed expression level difference between cells transfected with miRNA analogue and cells transfected with negative control (mock) oligonucleotides. The red legend in the bottom right corner of each plot shows the Pearson correlation coefficient of target effect between hsa-miR-299-3p transfection and ptr-miR-299-3p transfection into SH-SY5Y (panel A) and SK-N-SH (panel B) cell lines, calculated using Mock 1 as a negative control (R) and the correlation p-value (p). The X and Y-axis show the target effect produced by hsa-miR-299-3p and ptr-miR-299-3p transfection, respectively.
(TIF)

Table S1 This table lists tissue sample information.
(XLS)

Table S2 This table contains an overview of mapping statistics for small RNA Illumina sequencing data.
(XLS)

Table S3 This table lists miRNA expression levels in the prefrontal cortex and cerebellum of three species based on Illumina sequencing data.
(XLS)

Table S4 This table lists miRNA differently expressed between humans and chimpanzees and between humans and macaques in the prefrontal cortex identified using Fisher's exact test and edgeR package based on small RNA Illumina sequencing data.
(XLS)

Table S5 This table lists miRNA expression levels in prefrontal cortex of three species based on Agilent miRNA microarray data.
(XLS)

Table S6 This table lists miRNA with significant expression differences between humans and chimpanzees in the prefrontal cortex identified using small RNA Illumina sequencing and Agilent miRNA microarrays.
(XLS)

Table S7 This table contains protein expression levels measured in the prefrontal cortex of four humans and four chimpanzees.
(XLS)

Table S8 This table lists target effects of miRNA differently expressed between humans and chimpanzees in the prefrontal cortex at mRNA and protein levels.
(XLS)

Table S9 This table lists PANTHER biological process terms enriched in targets of miRNA with human-specific expression and in targets of miRNA with chimpanzee-specific expression.
(XLS)

Table S10 This table contains KEGG pathways enriched in targets of miRNA with human-specific expression.
(XLS)

Table S11 This table contains sequences of LNA-probes used in miRNA *in situ* hybridization experiments.
(XLS)

Table S12 This table lists target effects measured in five miRNA transfection experiments using nine miRNA target prediction tools.
(XLS)

Table S13 This table lists experimentally verified targets of miRNA based on our transfection experiments and the TargetScan predictions.
(XLS)

Table S14 This table lists experimental verified targets of miRNA based on our transfection experiments and predictions from all nine miRNA target prediction tools.
(XLS)

Table S15 This table contains PANTHER biological process terms and KEGG pathways enriched in experimentally verified targets of miRNA showing human-specific expression.
(XLS)

Table S16 This table contains GO terms and KEGG pathways enriched in experimental verified targets of miRNA showing human-specific expression, hsa-miR-34c-5p.
(XLS)
