# Supplementary figures and images for: MicroRNA Expression and Regulation in Human, Chimpanzee, and Macaque Brains
Source: PLoS Genet. 2011 Oct 13;7(10):e1002327. doi: 10.1371/journal.pgen.1002327 (PMC3192836; doi:10.1371/journal.pgen.1002327)

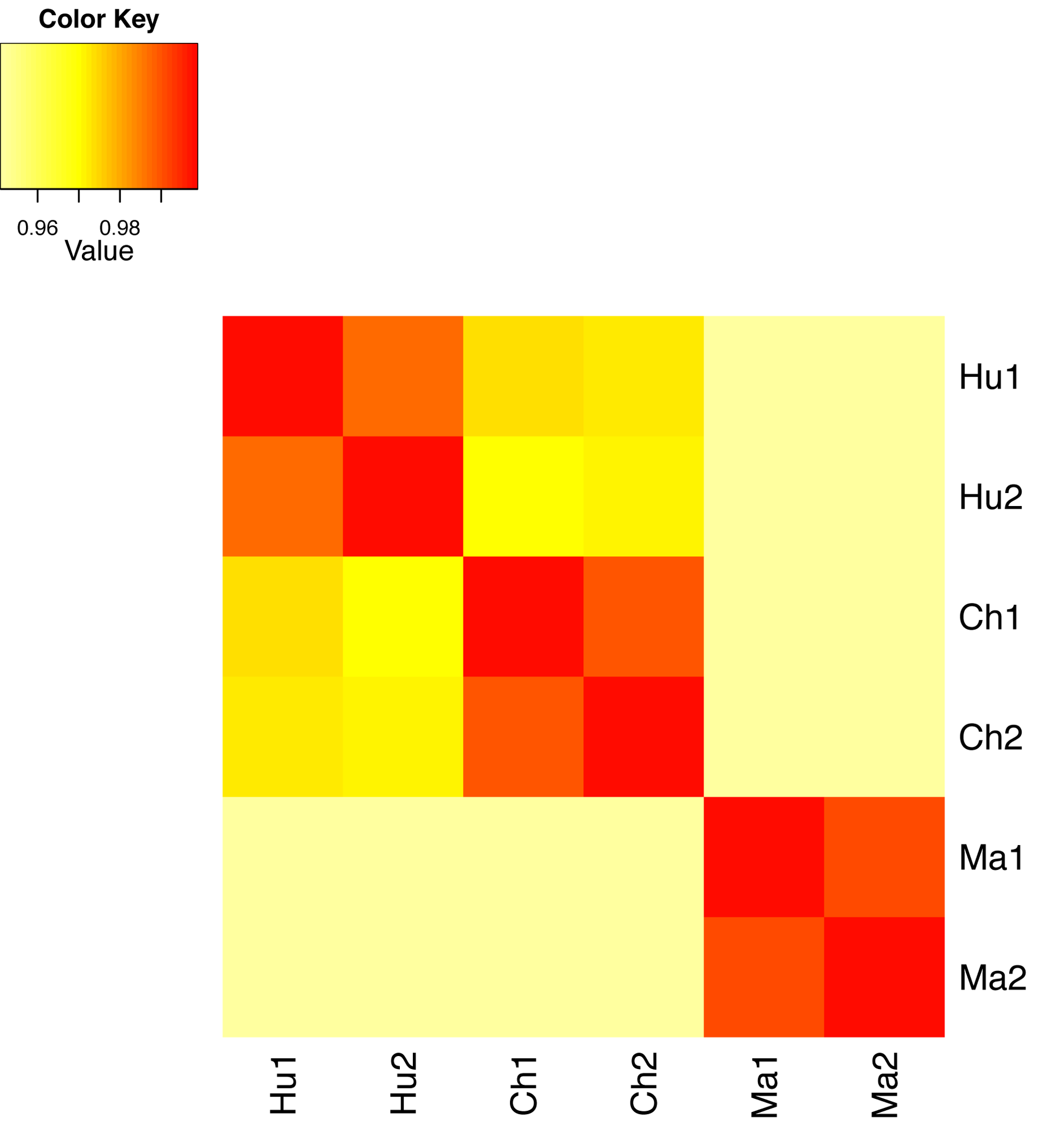

Supplement: Figure S1 — Correlations between miRNA expression profiles. The Pearson coefficients of miRNA expression level correlations between and within species based on 572 miRNA detected in the prefrontal cortex samples of all three species in small-RNA-seq data. The labels represent species: Hu – human; Ch – chimpanzee; Ma – rhesus macaque. (TIF) [file pgen.1002327.s001.tif]

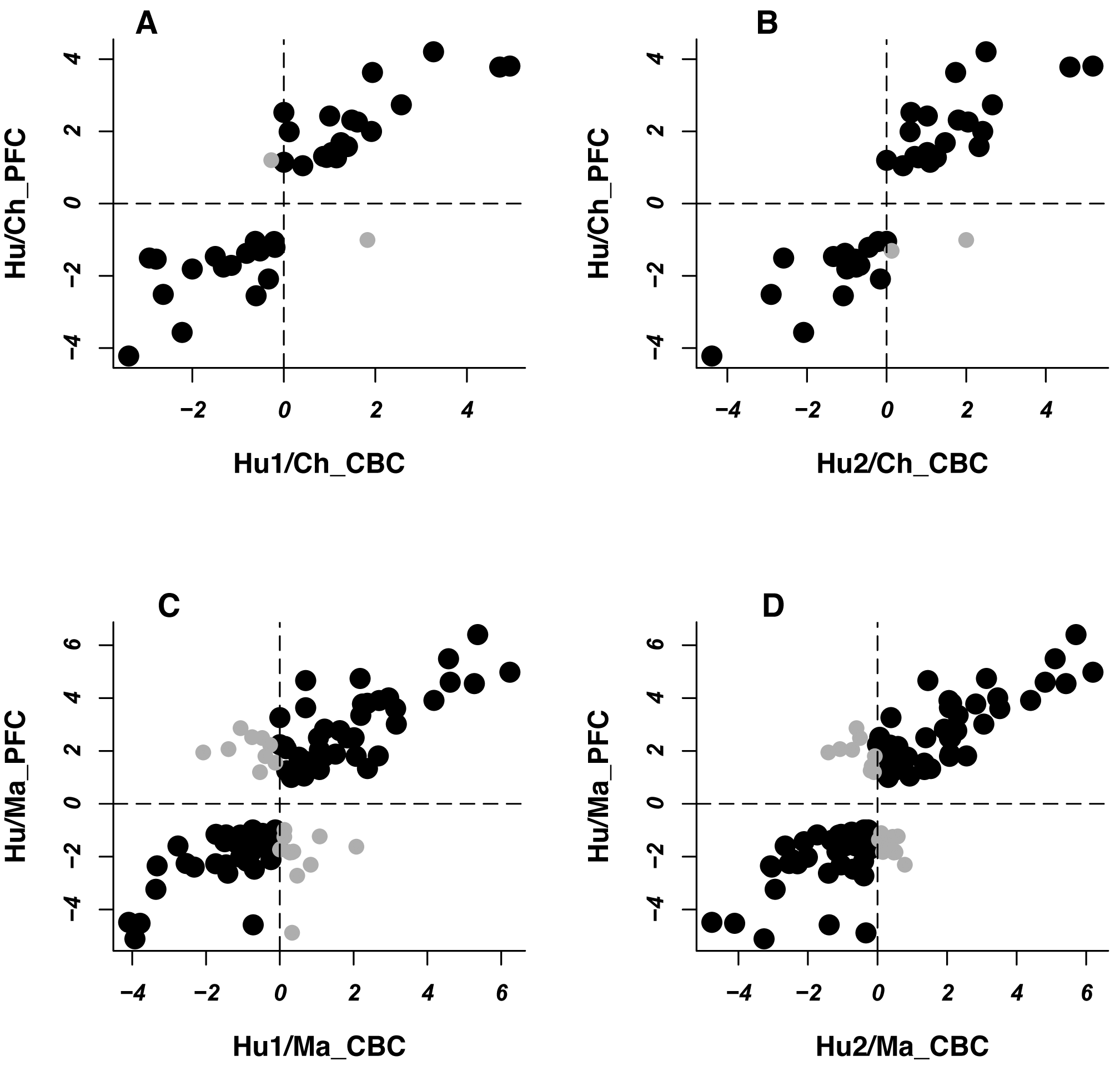

Supplement: Figure S2 — miRNA divergence between species measured in the prefrontal cortex and cerebellum. miRNA expression divergence (log2-transformed fold-changes) between humans and chimpanzees (N = 37) (A and B) or humans and rhesus macaques (N = 106) (C and D) measured using high-throughput sequencing in prefrontal cortex and cerebellum. The black dots indicate miRNA showing consistent direction of expression divergence in the two brain regions; grey dots – miRNA showing inconsistent directions of expression changes. The labels represent species: Hu – human; Ch – chimpanzee; Ma – rhesus macaque. Hu1 and Hu2 are two biological replicates of the human cerebellum sample. (TIF) [file pgen.1002327.s002.tif]

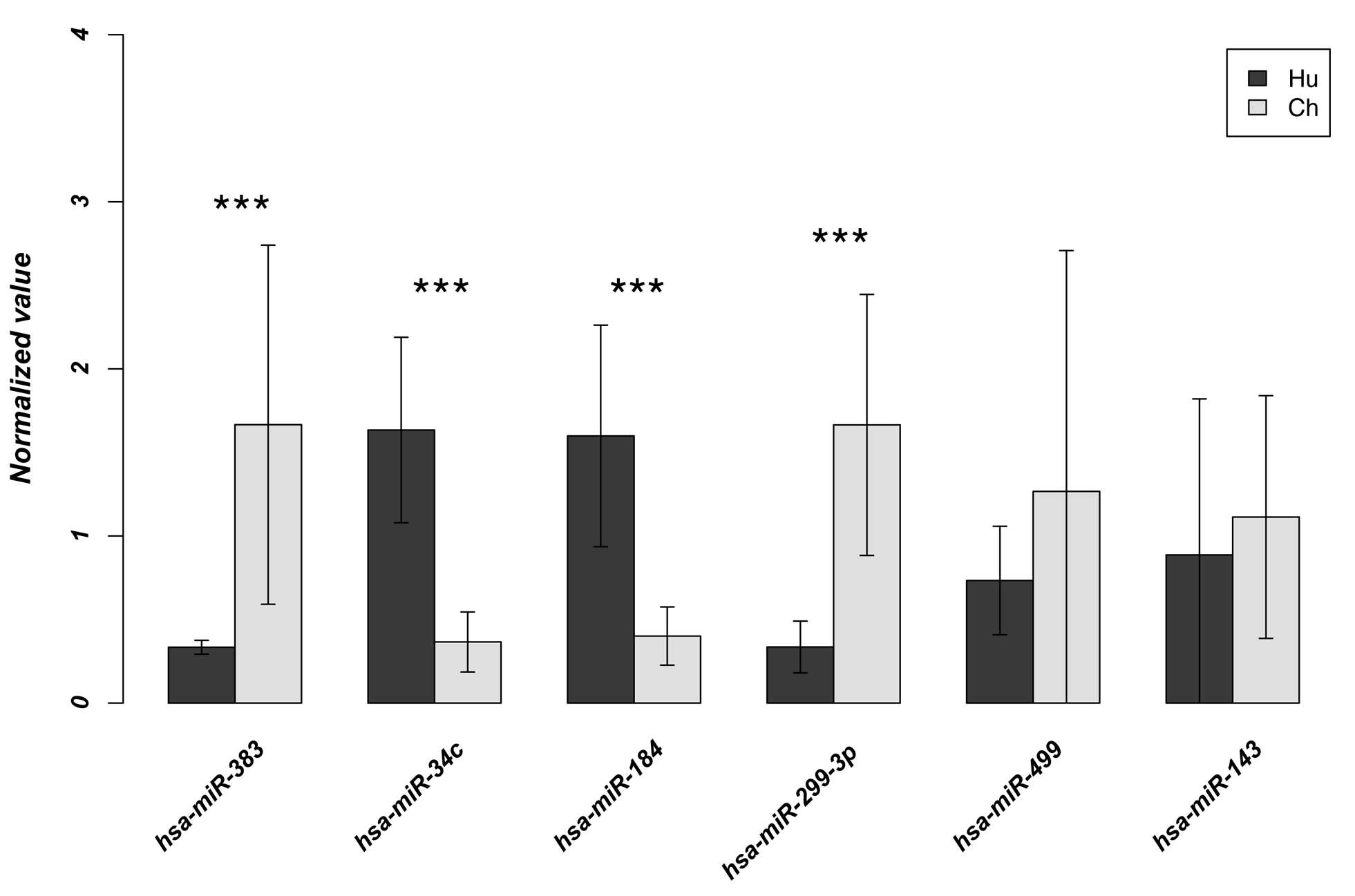

Supplement: Figure S3 — Q-PCR validation of species-specific miRNA expression. The histogram shows normalized miRNA expression levels in human (dark grey) and chimpanzee (light grey) prefrontal cortex samples measured using Q-PCR. The y-axis shows average miRNA expression normalized to the expression of invariant transcript (U6 snRNA). The error bars show one standard deviation based on duplicate Q-PCR experiments in three human and three chimpanzee samples. The symbols indicate significance levels: Student's t-test p<0.05 - *; p<0.01 - **; p<0.001 - ***. (TIF) [file pgen.1002327.s003.tif]

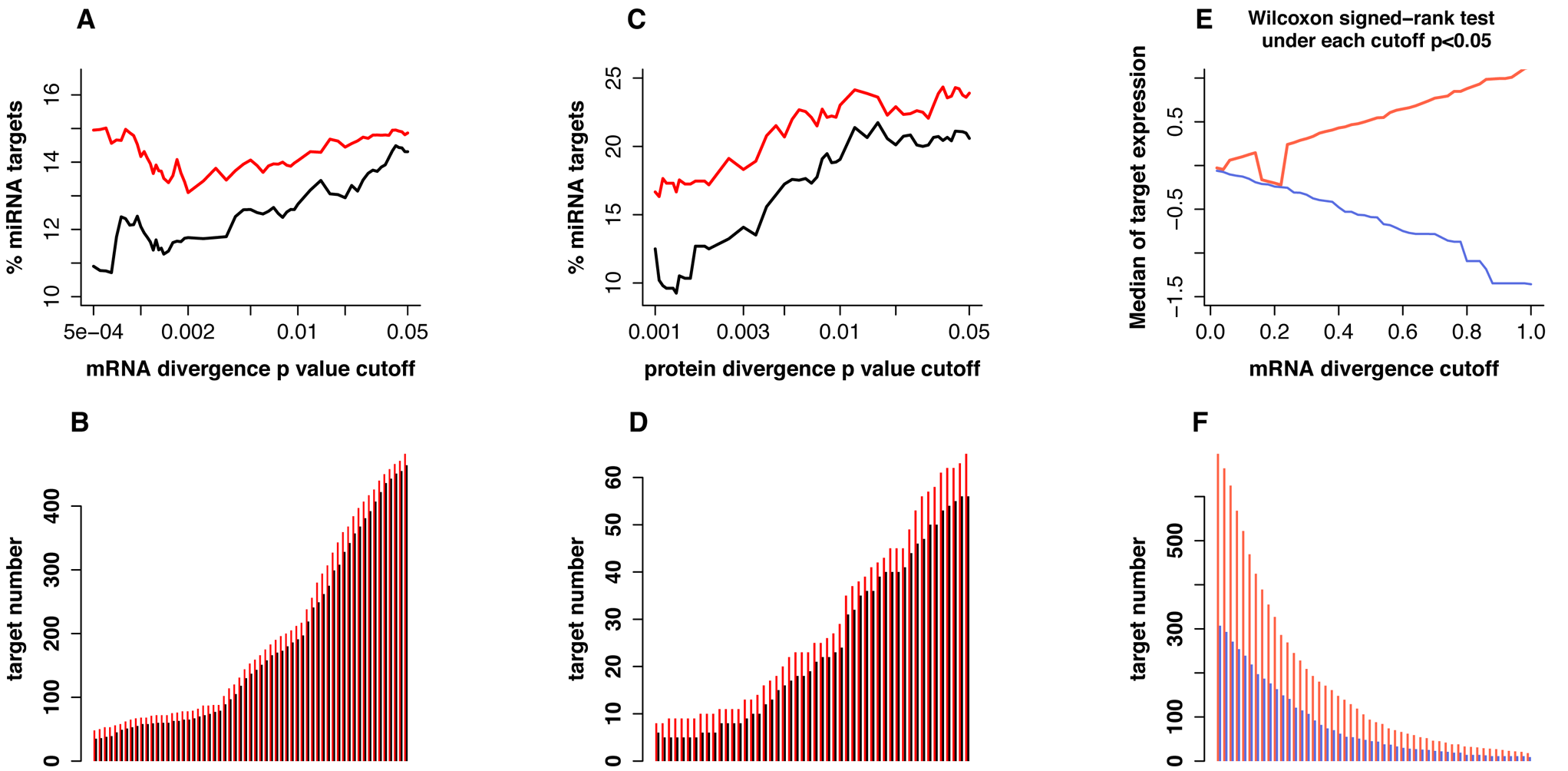

Supplement: Figure S4 — miRNA effect on mRNA and protein expression differences between human and chimpanzee prefrontal cortex (PFC). (A) Percentage of target mRNAs negatively (red curve) and positively (black curve) associated with 37 miRNAs differentially expressed in human and chimpanzee PFC at different mRNA divergence cutoffs. The mRNA divergence cutoff is based on Student's t-test p-values calculated using gene expression in human and chimpanzee individuals. (B) Numbers of target mRNA negatively (red bars) and positively (black bars) associated with 37 miRNAs differentially expressed in human and chimpanzee PFC at different mRNA divergence cutoffs shown in panel A. (C) Percentage of target proteins negatively (red curve) and positively (black curve) associated with 37 miRNAs differentially expressed in human and chimpanzee PFC at different protein divergence cutoffs. The protein divergence cutoff is based on Student's t-test p-values calculated using protein expression in human and chimpanzee individuals. (D) Numbers of target proteins negatively (red bars) and positively (black bars) associated with 37 miRNA differentially expressed in human and chimpanzee PFC at different protein divergence cutoffs shown in panel C. (E) Difference in median expression divergence between mRNA targeted by miRNA with high expression in the human PFC (blue curve), and mRNA targeted by miRNA with low expression in the human PFC (red curve), at different mRNA divergence cutoffs. Difference in median expression divergence between two mRNA groups was significant at each of the mRNA divergence cutoffs (Wilcoxon signed rank text, p<0.05). mRNA divergence cutoff was calculated based on absolute mean difference between human and chimpanzee expression levels. (F) Numbers of target genes for each mRNA divergence cutoff shown in panel E. (TIF) [file pgen.1002327.s004.tif]

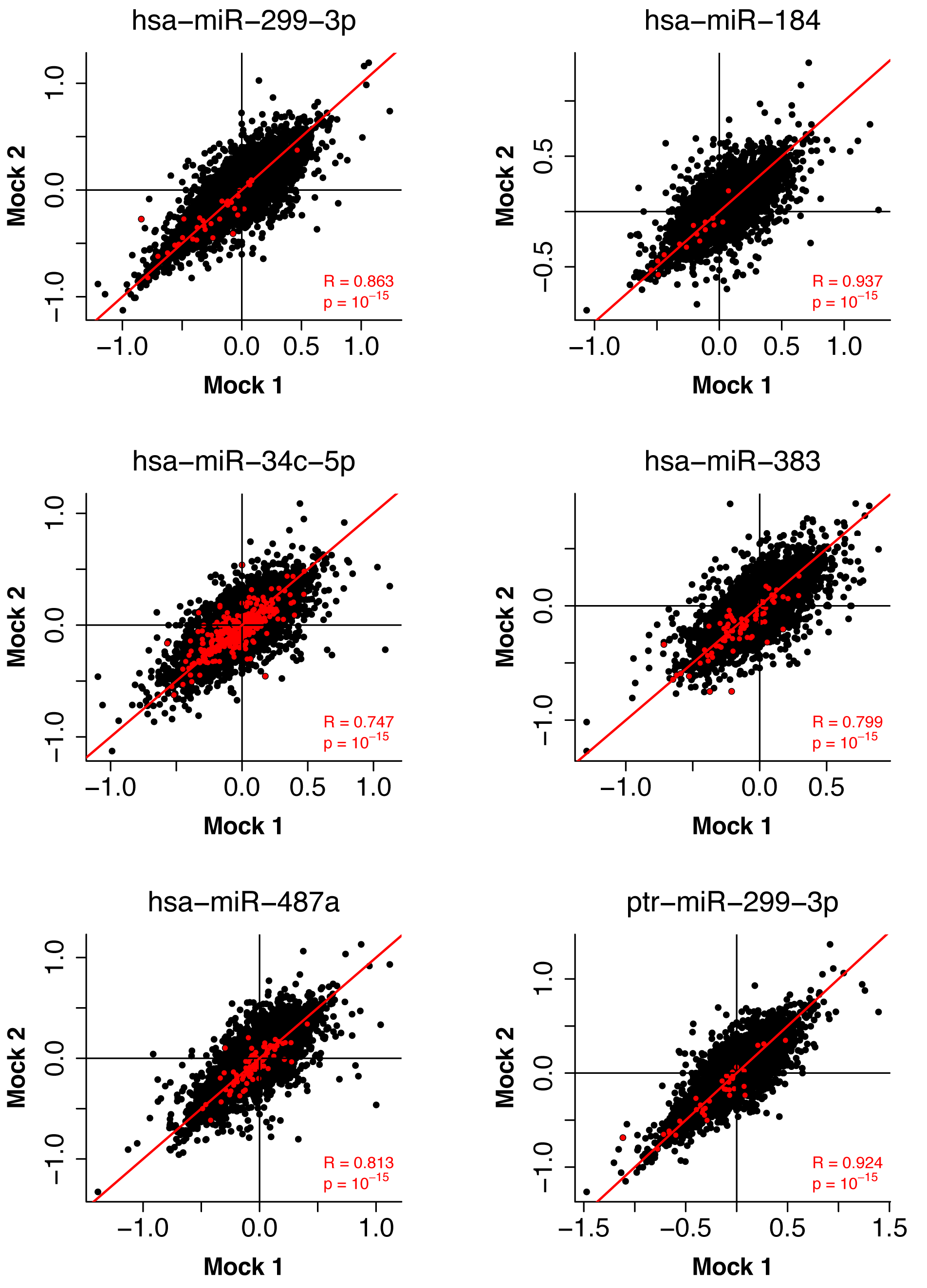

Supplement: Figure S5 — Target effect in two independent negative control replicates (Mock 1 and Mock 2) in SH-SY5Y cell line. The red points represent the target effect of conserved targets, predicted by TargetScan, with corresponding miRNA. The black points represent the background expression – all expressed genes, excluding targets of the miRNA, are shown. Target effect (miRNA regulatory effect) was calculated as the log2-transformed expression level difference between cells transfected with miRNA analogue and cells transfected with negative control (mock) oligonucleotides. The red legend in the bottom right corner of each plot shows the Pearson correlation coefficient of target effect using two negative controls (Mock 1 and Mock 2) in SH-SY5Y cell line (R) and correlation p-value (p). The X-axis shows the target effect using Mock 1 as a negative control, the Y-axis shows target effect using Mock 2 as a negative control. (TIF) [file pgen.1002327.s005.tif]

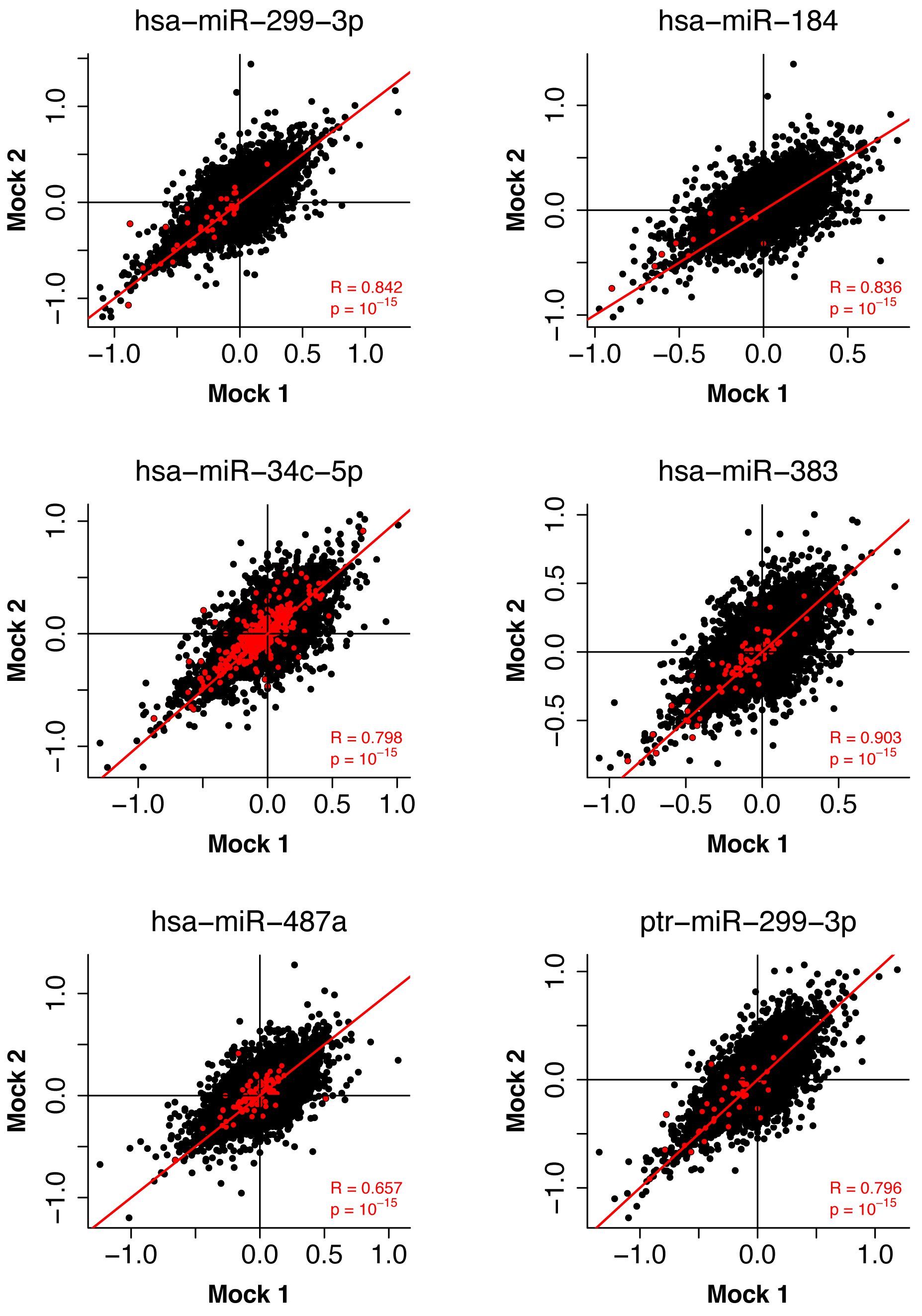

Supplement: Figure S6 — Target effect in two independent negative control replicates (Mock 1 and Mock 2) in SK-N-SH cell line. The red points represent the target effect of conserved targets, predicted by TargetScan, with corresponding miRNA. The black points represent the background expression – all expressed genes, excluding targets of the miRNA, are shown. Target effect (miRNA regulatory effect) was calculated as the log2-transformed expression level difference between cells transfected with miRNA analogue and cells transfected with negative control (mock) oligonucleotides. The red legend in the bottom right corner of each plot shows the Pearson correlation coefficient of target effect using two negative controls (Mock 1 and Mock 2) in SK-N-SH cell line (R) and correlation p-value (p). The X-axis shows the target effect using Mock 1 as a negative control, the Y-axis shows target effect using Mock 2 as a negative control. (TIF) [file pgen.1002327.s006.tif]

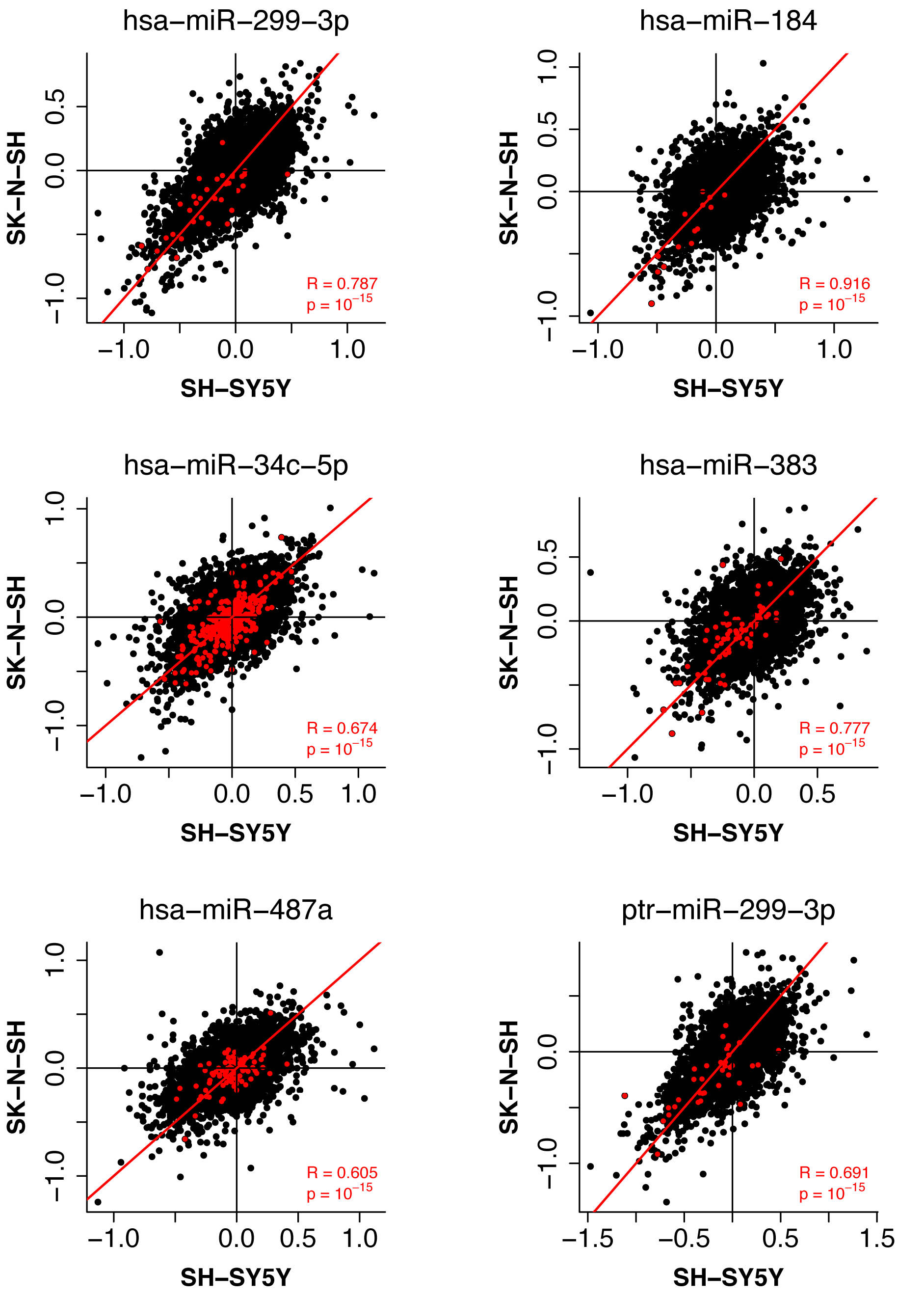

Supplement: Figure S7 — Target effect of miRNA transfection in two neuroblastoma cell lines. The red points represent the target effect of conserved targets, predicted by TargetScan, with corresponding miRNA. The black points represent the background expression – all expressed genes, excluding targets of the miRNA, are shown. Target effect (miRNA regulatory effect) was calculated as the log2-transformed expression level difference between cells transfected with miRNA analogue and cells transfected with negative control (mock) oligonucleotides. The red legend in the bottom right corner of each plot shows the Pearson correlation coefficient of the target effect between two cell lines, SH-SY5Y and SK-N-SH, calculated using Mock 1 as a negative control (R) and the correlation p-value (p). The X-axis shows target effect in SH-SY5Y cell line, the Y-axis shows target effect in SK-N-SH cell line. (TIF) [file pgen.1002327.s007.tif]

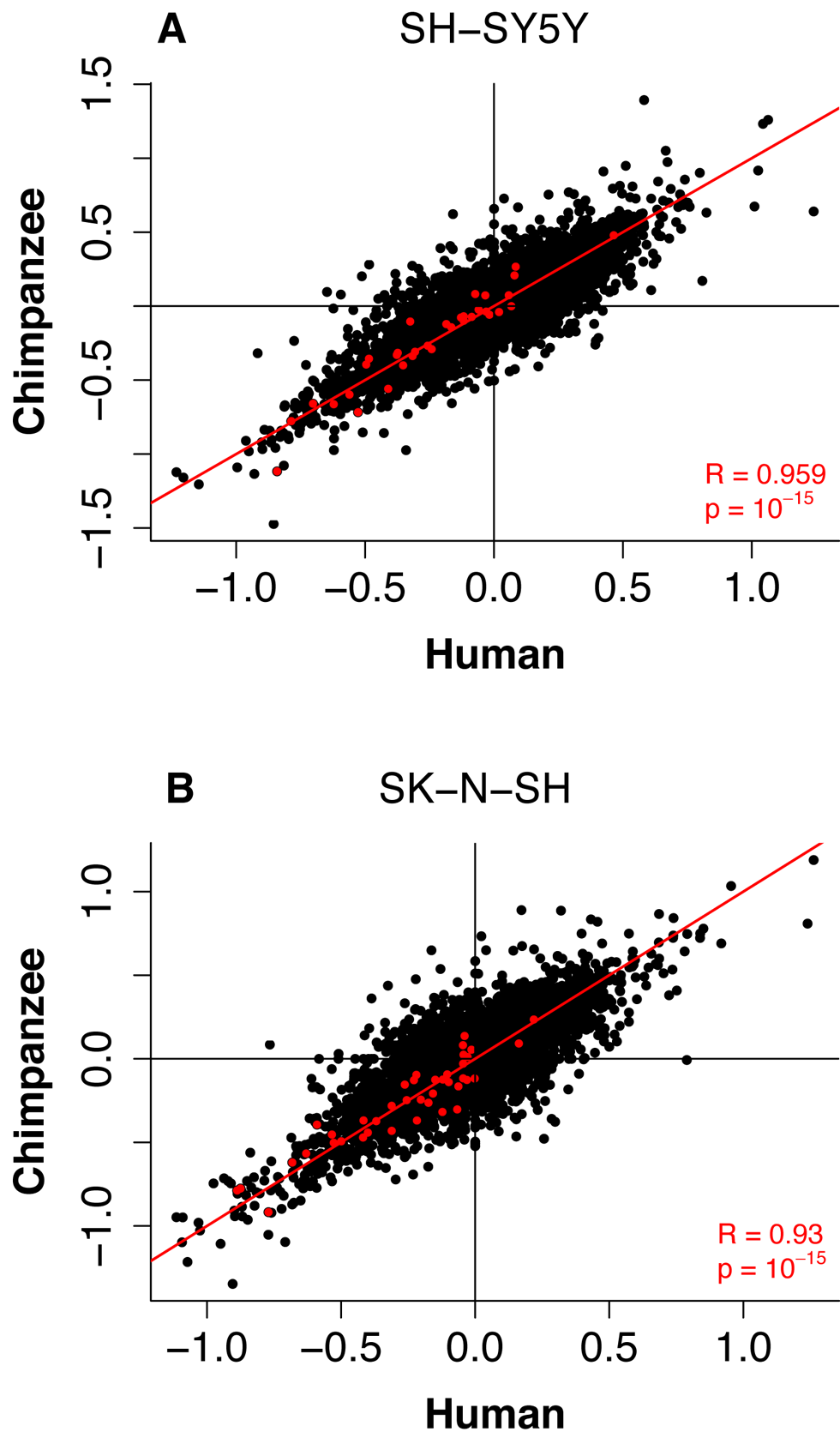

Supplement: Figure S8 — Target effects of human and chimpanzee variants of miRNA 299-3p (hsa-miR-299-3p and ptr-miR-299-3p) after transfection in two cell lines. The red points represent the target effect of conserved targets, predicted by TargetScan, with corresponding miRNA. The black points represent the background expression – all expressed genes, excluding targets of the miRNA, are shown. Target effect (miRNA regulatory effect) was calculated as the log2-transformed expression level difference between cells transfected with miRNA analogue and cells transfected with negative control (mock) oligonucleotides. The red legend in the bottom right corner of each plot shows the Pearson correlation coefficient of target effect between hsa-miR-299-3p transfection and ptr-miR-299-3p transfection into SH-SY5Y (panel A) and SK-N-SH (panel B) cell lines, calculated using Mock 1 as a negative control (R) and the correlation p-value (p). The X and Y-axis show the target effect produced by hsa-miR-299-3p and ptr-miR-299-3p transfection, respectively. (TIF) [file pgen.1002327.s008.tif]
